# Formation and Characterization of Xylitol-Modified Glycidyl Methacrylate-*co*-Ethyl Methacrylate Matrices for Controlled Release of Antimicrobial Compounds

**DOI:** 10.3390/molecules30153083

**Published:** 2025-07-23

**Authors:** Adam Chyzy, Przemysław Gnatowski, Edyta Piłat, Maciej Sienkiewicz, Katarzyna Wozniak, Marta Wojnicka, Krzysztof Brzezinski, Marta E. Plonska-Brzezinska

**Affiliations:** 1 Department of Organic Chemistry, Faculty of Medicine with the Division of Dentistry and Division of Medical Education in English, Medical University of Bialystok, Mickiewicza 2A, 15-222 Bialystok, Poland; adam.chyzy@sd.umb.edu.pl; 2 Department of Environmental Toxicology, Faculty of Health Sciences, Medical University of Gdańsk, Dębowa 23A, 80-204 Gdansk, Poland; przemyslaw.gnatowski@gumed.edu.pl; 3 Department of Polymer Technology, Faculty of Chemistry, Gdansk University of Technology, Narutowicza St. 11/12, 80-233 Gdansk, Poland; edyta.pilat@pg.edu.pl (E.P.); maciej.sienkiewicz@pg.edu.pl (M.S.); 4 Department of Structural Biology of Prokaryotic Organisms, Institute of Bioorganic Chemistry, Polish Academy of Sciences, Noskowskiego 12/14, 61-704 Poznan, Poland; kawozniak@ibch.poznan.pl (K.W.); mwojnicka@ibch.poznan.pl (M.W.); kbrzezinski@ibch.poznan.pl (K.B.)

**Keywords:** hydrogel, hydrophilic material, antibiotic, drug-delivery system, controlled drug release

## Abstract

Wounds are undeniably important gateways for pathogens to enter the body. In addition to their detrimental local effects, they can also cause adverse systemic effects. For this reason, developing methods for eradicating pathogens from wounds is a challenging medical issue. Polymers, particularly hydrogels, are one of the more essential materials for designing novel drug-delivery systems, thanks to the ease of tuning their structures. This work exploits this property by utilizing copolymerization, microwave modification, and drug-loading processes to obtain antibacterial gels. Synthesized xylitol-modified glycidyl methacrylate-*co*-ethyl methacrylate ([P(EMA)-*co*-(GMA)]-Xyl]) matrices were loaded with bacitracin, gentian violet, furazidine, and brilliant green, used as active pharmaceutical ingredients (APIs). The hydrophilic properties, API release mechanism, and antibacterial properties of the obtained hydrogels against *Escherichia coli*, *Pseudomonas aeruginosa*, and *Staphylococcus epidermidis* containing [P(EMA)-*co*-(GMA)]-Xyl] were studied. The hydrogels with the APIs efficiently inhibit bacteria growth with low doses of drugs, and our findings are statistically significant, confirmed with ANOVA analysis at *p* = 0.05. The results confirmed that the proposed system is hydrophilic and has extended the drug-release capabilities of APIs with a controlled burst effect based on [P(EMA)-*co*-(GMA)]-Xyl] content in the hydrogel. Hydrogels are characterized by the prolonged release of APIs in a very short time (a few minutes). Although the amount of released APIs is about 10%, it still exceeds the minimum inhibitory concentrations of drugs. Several kinetic models (first-order, second-order, Baker–Lonsdale, and Korsmeyer–Peppas) were applied to fit the API release data from the [P(EMA)-*co*-(GMA)]-Xyl-based hydrogel. The best fit of the Korsmeyer–Peppas kinetic model to the experimental data was determined, and it was confirmed that a diffusion-controlled release mechanism of the APIs from the studied hydrogels is dominant, which is desirable for applications requiring a consistent, controlled release of therapeutic agents. A statistical analysis of API release using Linear Mixed Model was performed, examining the relationship between % mass of API, sample (hydrogels and control), time, sample–time interaction, and variability between individuals. The model fits the data well, as evidenced by the determination coefficients close to 1. The analyzed interactions in the data are reliable and statistically significant (*p* < 0.001). The outcome of this study suggests that the presented acrylate-based gel is a promising candidate for developing wound dressings.

## 1. Introduction

Skin is the human body’s largest organ, with a surface area of 1.5 and 2 m^2^, accounting for approximately 16% of the human body mass. Its thickness depends on the location, usually ranging from 0.5 to 4 mm, thickest on the heels and thinnest around the eyes, especially on the eyelids [1]. The skin has many functions related to its structure, but the most important one is that it is a protective barrier to the body. For instance, several layers of keratinocytes covered by a lipid layer act as a semi-permeable barrier against the penetration of pathogenic microorganisms. In addition, skin also plays a role in the synthesis of vitamin D_3_, immune functions, and body-temperature regulation through the control of water transpiration [2,3,4]. Additionally, the skin has its unique microbiome, which is acquired at birth and evolves throughout life. Its composition is strictly dependent on the skin area and environmental conditions. The natural commensals of human skin present under physiological conditions include various bacteria such as *Propionibacterium* spp., *Staphylococcus* spp., *Corynebacterium* spp., as well as viruses such as *Human Papillomavirus* or *Herpes simplex* and fungi such as *Malassezia* spp., *Aspergillus* spp., *Cryptococcus* spp. [5]. However, when the skin is injured, numerous human pathogens can bypass the natural body barrier, leading to infections or dermatoses [6]. Dermatoses can be caused by multiple pathogenic microorganisms, including Gram-positive bacteria such as staphylococci (e.g., *Staphylococcus aureus* or *Methicillin-resistant Staphylococcus aureus* (MRSA)), streptococci (e.g., *Streptococcus pyogenes*), mycobacteria (e.g., *Corynebacterium minutissimum*), clostridia (e.g., *Clostridium tetani* or *C. perfringens*), and Gram-negative bacteria (e.g., *Escherichia coli*, *Proteus* spp. and *Pseudomonas* spp.).

Dermatoses are usually treated by semi-solid topically administered forms of drugs, which can be differentiated into four groups depending on their base type [7]. They can be based on emulsions: oil-in-water utilized in water-washable creams or water-in-oil used in absorption bases [8]. On the other hand, creams with oleaginous bases are not easily removed with water and are usually prepared with hydrocarbons. Gels are the last distinguished group, made from hydrophilic components. If the gel has a hydrophilic solvent medium, it is called a hydrogel, whereas a hydrophobic medium, e.g., oil, is an oleogel component [9].

Physically, gels are a spatial system often referred to as a three-dimensional structure formed by long-chain polymers with non-structured solvent (usually water) molecules diving between them. Since water molecules and polymer chains differ significantly from the chemical point of view, hydrogels are classified as heterogels. This pharmaceutical dosage form is widely used nowadays in modern therapies against dermatological diseases [10,11,12]. Additionally, hydrogels have been used extensively in wound treatment as dressings [13,14,15,16]. An interesting group of hydrogels with a promising potential for therapeutic use are their injectable forms [17,18,19,20]. Hydrogels can be used as vehicles for the delivery of substances with antifungal [21,22,23], antimicrobial [24,25,26,27,28] and anticancer [29,30,31,32] activities.

One of the most attractive polymers for hydrogel formations are acrylates and methacrylates. The simplicity of chemical modifications of these polymers makes it possible to modulate the mechanical strength, water retention, and thermal stability of the resulting materials [33,34]. Various studies show that they can successfully be used in tissue engineering or cell culture [35,36,37,38,39]. In particular, glycidyl methacrylate (GMA) and ethyl methacrylate (EMA) are widely used to form various polymers with high mechanical resistance, flexibility, and transparency [40,41]. A feature distinguishing GMA from other methacrylates is the presence of the highly reactive oxiran moiety in its structure. Incorporating hydrophilic molecules into the oligomeric chain increases water solubility, a feature necessary for gel formation.

A novel polymeric matrix based on EMA and GMA oligomers for efficient and targeted antibacterial drug delivery was developed to address the need for advanced biomaterials for dermatosis treatment. Herein, we describe the physicochemical and antibacterial properties of the hydrogel materials based on agarose (AGR) and the xylitol (Xyl)-functionalized oligomers containing EMA and GMA [P(EMA)-*co*-(GMA)]-Xyl] [42]. Within our research, we have tested the obtained materials as a potential drug-release system for four active pharmaceutical ingredients (APIs) with antibacterial properties, namely bacitracin (BAC) [43,44,45,46], gentian violet (GV) [47,48,49], furazidine (FUR) [50], and brilliant green (BG) [51,52,53]. APIs with different hydrophobic–hydrophilic properties were used to study the interaction of the [P(EMA)-*co*-(GMA)]-Xyl] matrix with the released substances. Additionally, stability, the hydrophilic nature of the matrix, and the kinetic parameters of API release processes were assessed. Several kinetic models (first-order, second-order, Baker–Lonsdale, and Korsmeyer–Peppas) were applied to fit the API release data from the [P(EMA)-*co*-(GMA)]-Xyl/AGR-based hydrogel. A statistical analysis of microbiological studies and API release using Linear Mixed Model (LMM) were used to examine the relationship between % mass of API, sample (hydrogels and control), time, sample–time interaction, and variability between individuals. The study demonstrates novelty through its unique integration of short hydrophobic EMA and GMA oligomers that are stabilized with AGR and functionalized with Xyl to develop a hydrophilic matrix for antibacterial drug-delivery applications.

## 2. Results and Discussion

### 2.1. The Synthesis of P(EMA)-co-(GMA)]-Xyl and the Preparation of Drug-Loaded Gels for Microbiological and API Release Studies

The synthesis of the cooligomers containing EMA and GMA was performed giving network [P(EMA)-*co*-(GMA)]) using our previously published procedure [42]. The preparation of methacrylic acid ester oligomers/polymers is based on a radical polymerization reaction initiated with azobisisobutyronitrile (AIBN) [54]. The reaction was carried out in a heptane solution to reduce the dispersion of the obtained product—oligomer, and to facilitate the use of precipitation polymerization. A [P(EMA)-*co*-(GMA)] oligomer was obtained in which the ratio of EMA to GMA units in the chain was 1:2.5. To change the hydrophobic character of the [P(EMA)-*co*-(GMA)] to a more hydrophilic one, the oligomeric chain was functionalized with several modifiers, exhibiting hydrophilic properties due to the presence of numerous -OH groups in the structure. Cytotoxicity tests were performed for a series of modified cooligomers, and only for the Xyl-functionalized [P(EMA)-*co*-(GMA)] low cytotoxicity was recorded.

[P(EMA)-*co*-(GMA)]-Xyl was obtained as a result of the reaction between [P(EMA)-*co*-(GMA)] and Xyl in anhydrous dimethylformamide. For successful functionalization of [P(EMA)-*co*-(GMA)] in post-polymerization reaction, 1,8-diazabicyclo(5.4.0)undec-7-ene was used as a strong base to deprotonate the -OH group of Xyl [55]. In our previous work, the synthesized oligomeric materials were characterized, and a cytotoxicity test was performed, and the swelling factor was evaluated. [P(EMA)-*co*-(GMA)] and modified [P(EMA)-*co*-(GMA)]-Xyl showed the best performances. Human-skin fibroblasts exposed to extracts of the mentioned cooligomers exhibited high viability, near 100%, throughout the experiment [42]. Therefore, in our studies presented in this paper, we used only [P(EMA)-*co*-(GMA)]-Xyl to form the hydrogel matrix and performed microbiological and in vitro release studies.

The cooligomers have short chains, consisting of less than 10 units in a chain, which significantly influence the structure of the matrix of the formed hydrogel. Molecular mass distribution for [P(EMA)-*co*-(GMA)]-Xyl, in the range from 601.2 to 300.8 Da, is 65.7% of the total cooligomer mass with an average molar mass of 436.50 [42], which is why AGR was added to improve the properties of the formed cooligomer matrix. Two different mass ratios of ([P(EMA)-*co*-(GMA)]-Xyl) to AGR were used to prepare hydrogels, as presented in Table 1. Gel 1 with [P(EMA)-*co*-(GMA)]-Xyl had a AGR mass ratio of 2:1, and Gel 2 with [P(EMA)-*co*-(GMA)]-Xyl had a AGR mass ratio of 1:1. In our studies, we used both gels with different properties to investigate the in vitro release and the possible interaction of the cooligomer matrix with the APIs, the influence of the API size, and other factors that may affect the practical use of the proposed hydrogel.

### 2.2. An Influence of API-Loaded [P(EMA)-co-(GMA)]-Xyl/AGR Gels on Bacterial Growth

**Figure 1:**
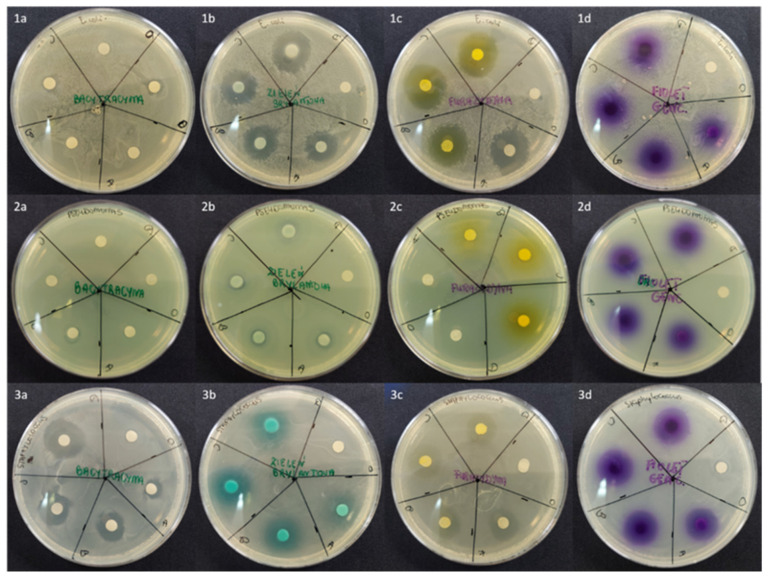
The antimicrobial activity of API-loaded Gel 2 against (**1**) *E. coli*, (**2**) *P. aeruginosa*, (**3**) *S. epidermidis*. Gels loaded with (**a**) BAC, (**b**) BG, (**c**) FUR, (**d**) GV. The concentration of API (markings on dishes): (0) control, (A) 0.1%, (B) 0.5%, (C) 1.0%, (D) 2.0%. For each bacterium–API pair, the analysis was performed in duplicate. Bacteria growth inhibition zones in parentheses correspond to API-loaded gel without the addition of [P(EMA)-*co*-(GMA)]-Xyl. Bacteria growth inhibition zones are averaged and are given with standard deviations.

Bacteriological tests performed for API-loaded Gel 2 indicated that the antimicrobial activity of used APIs is preserved during the preparation process (Table 1 and Table 5). However, various effects were observed for tested formulations. Generally, Gel 2 revealed similar or higher antimicrobial properties compared to the control gel (AGR, Table 1). Zones of inhibition for each API-loaded Gel 2 are shown in Figure 1. No significant or minor differences between gel types were found for such pairs of API-bacterium as BG-*E. coli*, GV-*P. aeruginosa*, FUR-*S. epidermidis*, BG-*S. epidermidis*, and GV-*S. epidermidis*.

**Table 1:** The diameters of the bacteria growth inhibition zones observed depending on the API type and loading amount in Gel 2 and control (in brackets) gels.

Microbial Strain	API	Bacteria Growth Inhibition Zone (mm)			
		A (0.1%)	B (0.5%)	C (1.0%)	D (2.0%)
*Escherichia coli* DH5α	BAC	Not observed			
	BG	16.5 ± 0.7 (16.5 ± 2.1)	17.5 ± 0.7 (18.5 ± 2.1)	19.5 ± 0.7 (18.0 ± 1.4)	20.5 ± 2.1 (20.0 ± 1.4)
	FUR	20.5 ± 0.7 (18.5 ± 0.7)	21.5 ± 0.7 (19.0 ± 1.4)	20.5 ± 2.1 (18.0 ± 1.4)	21.0 ± 1.4 (19.0 ± 1.4)
	GV	10.0 ± 1.4 (8.5 ± 0.7)	11.5 ± 0.7 * (6.5 ± 0.7)	12.5 ± 0.7 * (6.0 ± 1.4)	13.0 ± 1.4 * (7.0 ± 1.4)
*Pseudomonas aeruginosa* ATCC 9027	BAC	Not observed			
	BG	10.0 ± 1.4 (8.5 ± 0.7)	11.5 ± 0.7 (8.0 ± 1.4)	13.0 ± 0.0 (9.0 ± 1.4)	13.0 ± 1.4 (8.5 ± 2.1)
	FUR	Not observed			
	GV	7.5 ± 0.7 (8.5 ± 2.1)	9.0 ± 1.4 (8.0 ± 1.4)	9.5 ± 0.7 (9.5 ± 0.7)	10 ± 1.4 (9.0 ± 1.4)
*Staphylococcus epidermidis* ATCC 12228	BAC	7.5 ± 0.7 (9.0 ± 1.4)	15.0 ± 1.4 ** (12.0 ± 1.4)	17.0 ± 1.4 ** (14.5 ± 0.7)	19.5 ± 2.1 ** (17.5 ± 2.1) **
	BG	14.0 ± 1.4 (8.5 ± 0.7)	17.0 ± 1.4 (12.5 ± 0.7)	19.0 ± 1.4 (16.0 ± 1.4) **	20.5 ± 2.1 ** (16.5 ± 2.1) **
	FUR	13.5 ± 0.7 (13.0 ± 1.4)	15.5 ± 0.7 (15.5 ± 0.7)	16.5 ± 2.1 (15.5 ± 2.1)	17.5 ± 0.7 (18.0 ± 1.4)
	GV	16.0 ± 0.0 (15.5 ± 0.7)	16.5 ± 0.7 (15.5 ± 2.1)	17.5 ± 2.1 (16.5 ± 0.7)	19.0 ± 1.4 (17.0 ± 1.4)

API: bacitracin (BAC), gentian violet (GV), furazidine (FUR), brilliant green (BG). * Result statistically different from corresponding control sample result at *p* < 0.05. ** Result statistically different from corresponding sample with 0.1% API loading result at *p* < 0.05.

For FUR-*E. coli* (slightly) and GV-*E. coli* (significantly, confirmed with ANOVA analysis at *p* = 0.05), and BG-*S.epidermidis* and BG-*P. aeruginosa* (slightly) pairs, a more potent inhibition of bacterial growth was observed for Gel 2 with the addition of [P(EMA)-*co*-(GMA)]-Xyl]. A slight dose-dependent effect is observed for Gel 2 for the latter pair. Interestingly, statistically significant differences at some API concentrations were observed for BAC-*S. epidermidis* and BG-*S. epidermidis* pairs, where a dose-dependent effect is observed for the tested gel and the control. However, remarkably stronger growth inhibition is observed for Gel 2, as the diameter of the inhibition zones was larger by 2 and 4 mm, respectively. Finally, no or modest antimicrobial effects were observed for the tested gels and controls for the following pairs: BAC-*E. coli*, BAC-*P. aeruginosa*, and FUR-*P. aeruginosa*. The larger inhibition zones for FUR can be associated with the solubility of the substance. FUR is more easily released from Gel 2 as a hydrophobic substance. Hydrophilic substances (BAC, BG, GV) have a more hindered ability to permeate the bacterial wall and membranes. However, the results show that Gel 2 causes a higher inhibition than control. All results lead to the conclusion that the [P(EMA)-*co*-(GMA)]-Xyl-based gel formulations can be considered effective carriers of antibacterial drugs for external application.

### 2.3. Hydrophilic Properties of [P(EMA)-co-(GMA)]-Xyl Cooligomer

The contact angle of the [P(EMA)-*co*-(GMA)]-Xyl cooligomer was determined. Measurements were carried out for two polar liquids (water and glycerol) and one non-polar—diiodomethane. Table 2 shows the mean value of the contact angle defined in the tests with the standard deviation and photographs of the individual measurements, which are presented in Figure S1. The calculated total surface free energy of the material was determined to be 45.3 ± 0.1 mJ m^−2^, and the Lifshitz–van der Waals (dispersive), acid–base, acid (electron acceptor), and base (electron donor) components of energy of solid were as follows: 48.20 ± 0.02, −2.90 ± 0.11, 0.10 ± 0.01, and 21.40 ± 0.19 mJ m^−2^.

Contact-angle testing showed that the [P(EMA)-*co*-(GMA)]-Xyl-based gel has hydrophilic properties with a noticeable basic character. The calculated surface energy value shows that the obtained cooligomer can be classified as a medium surface energy material. Its surface energy is comparable to the surface energy of nylon, polyester, or epoxy material, with the latter comparison confirming the origin of the material obtained. These surface properties suggest a moderately hydrophobic material capable of engaging in specific intermolecular interactions, particularly hydrogen bonding. Such a profile is advantageous in drug-delivery systems, where interactions with biologically active compounds are essential for controlled release.

The rheological properties of Gel 1 were analyzed in terms of temperature and frequency sweep, which are presented in Figure S2 and Table S1. During the temperature sweep measurement, a slight decrease in both the storage modulus (*G*’) and the loss modulus (*G*”) was noticed with increasing temperature. In the case of the frequency sweep, the most significant observation was made at a frequency of approx. 100 Hz, were the *G*’ and *G*” curves intersected, indicating a gel-to-sol transition. Based on these results, it can be concluded that Gel 1, which showed a higher mass fraction of [P(EMA)-*co*-(GMA)]-Xyl to AGR (mass ratio of 2:1), remains mechanically stable, as only minor changes in *G’* and *G*” were observed within the temperature range of 20–40 °C and frequency range of 0.1–50 Hz—conditions under which these materials are expected to be applied.

Maintaining adequate mechanical strength at high water content, crucial for the functionality of materials in biomedical applications, depends on the degree of polymer crosslinking and the type of monomers used. Therefore, the higher water content of the gel material with adequately preserved mechanical resistance offers the possibility of a wider use of this material as a drug carrier, especially for hydrophilic substances [56,57].

### 2.4. BG Degradation Studies in Relation to Light, Temperature, and Solvent

The performed BG degradation studies are summarized in Table 3. It was noticed that the systems with PBS as an acceptance solution were characterized by significantly lower concentrations of BG, indicating the decomposition of this API. In comparison, the samples incubated in water showed a much more minor, almost negligible decrease in absorbance. For this reason, the study of the release of the API from the BG-loaded [P(EMA)-*co*-(GMA)]-Xyl/AGR gel was performed using water as the release medium. Additionally, it was observed that in a water medium, the light and temperature exposition did not significantly affect BG concentration.

### 2.5. In Vitro API Release Study

To investigate the influence of the obtained ([P(EMA)-*co*-(GMA)]-Xyl) on the release of APIs, two different mass ratios of the cooligomer and AGR were used, as presented in Table 5. Gel 1 with [P(EMA)-*co*-(GMA)]-Xyl to AGR mass ratio of 2:1, and Gel 2 with a mass ratio of 1:1. This approach allows us, among other things, to observe the relationship between the size of the released drug molecule and the cross-linking of the hydrogel’s matrix, and interaction of APIs with the gel formulation and their hydrophilicity. MiliQ water was used as the release medium for BG, whereas PBS was used for other APIs due to observed discoloration of the BG solution during initial tests. The exact values of released APIs are presented in Table S2.

**Figure 2:**
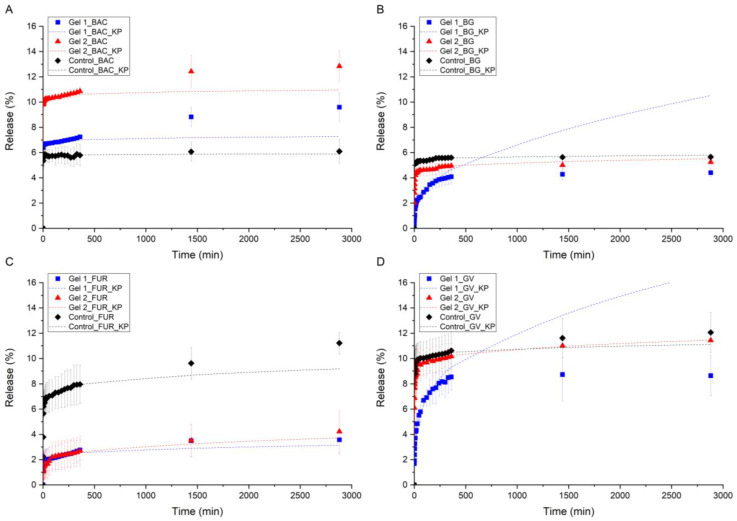
Percentages of API released in time (symbols) and fitted Korsmeyer–Peppas kinetic model (lines): (**A**) BAC, (**B**) BG, (**C**) FUR, and (**D**) GV. Blue lines and squares refer to Gel 1, red lines and triangles to Gel 2, and black lines and diamonds to the control sample.

In vitro release data has shown a two-phase cumulative release profile of BAC, BG, GV, and FUR for all the formulations, burst release in the first stage, and diffusion-mediated release [58]. Generally, for both hydrogels (Gel 1 and Gel 2), an instant-burst drug release was observed during the first 15 min, followed by a stabilized, slow release over the next 5 h, reaching equilibrium release, with some exceptions (Figure 2). The burst effect in in vitro drug-release studies refers to the rapid release of a significant portion of the drug from a delivery system shortly after administration [59]. It is strongly related to the physical properties of the drug formulation and the interactions between the drug and the surrounding environment (matrix and solution) [60]. It should also be emphasized that the matrix used, [P(EMA)-*co*-(GMA)]-Xyl/AGR, has amphiphilic properties, while the cooligomer has a hydrophobic segment P(EMA)-*co*-(GMA), which may depress the penetration of water into the polymeric matrix, which may also enhance the burst effect in our studied system [60].

The release profiles of BAC (Figure 2A, Table S2) from Gel 1 and Gel 2 are similar, with a mild burst effect in the first minutes followed by a slow, steady release throughout the experiment. The amount of released BAC (also BG and GV) is higher for Gel 2, which the lower cooligomer content in hydrogel can explain. AGR, which is an additive in gels involved in the formation of a denser and a more cohesive network (Gel 1), limiting the diffusion of BAC through the hydrogel matrix ([P(EMA)-*co*-(GMA)]-Xyl/AGR), providing a slower release. Both Gel 1 and Gel 2 are characterized by prolonged release of BAC, whereas in the control sample (AGR), a burst effect without further release of API was observed. This analysis shows the influence of the particle size of the drug in the tested hydrogel’s matrix very well, highlighting the mass transport through the gel. BAC is a large molecule with a molecular weight of 1422.71 g mol^−1^, the largest of our studied APIs. Therefore, the observed BAC release trend differs from other APIs (Figure 2A). Only for this molecule, release from the control matrix (AGR) is confining, indicating a substantial diffusion limitation from the matrix, characterized by the lowest release value. In the case of the gel with the highest density and the cross-linking of the polymer (control matrix—AGR), BAC has difficulty diffusing from the matrix to the solution, which is revealed by the lowest degree of drug release from the matrix at the level of about 5%. The amount of released BAC increases with the increase in the amount of cooligomer in the matrix, observed with the highest value for Gel 2, reaching twice the value of drug release compared to the control gel.

In the case of BG release (Figure 2B, Table S2), it can be seen that only a small amount of the API is released from all three gels (Gel 1, Gel 2, and AGR) through the experiment period, indicating a substantial limitation of diffusion. In the case of Gel 2 and the control sample, rapid burst release was observed during the first minutes of the experiment, quickly reaching the equilibrium. Gel 1 was characterized as a significantly slower release profile, reaching the equilibrium after the 6th hour of the experiment. Despite its good solubility, BG is effectively retained in the hydrogel matrix, probably due to the interactions with both components of the matrix ([P(EMA)-*co*-(GMA)]-Xyl/AGR). Adding twice the oligomer compared to AGR (Gel 1) resulted in a much more controlled and gradual release of the hydrophilic API from the cooligomer-containing matrix. It should be noted, however, that the total amount released was lower than in the case of the other two hydrogels (Gel 2 and AGR).

The profiles of the drug release from the matrix in the case of GV (Figure 2D, Table S2) were similar to BG, with the difference that the amount of released substance is twice as high in the case of GV. In Gel 1 with GV, release occurs gradually, without the evident burst release. It indicates that a continuous diffusion gradient is maintained, and GV penetration through the matrix is effectively reduced. The structure of [P(EMA)-*co*-(GMA)]-Xyl/AGR allows for a constant, prolonged release, which is desirable for designing an API-controlled release system. On the other hand, in Gel 2, GV is released much faster, with a strong burst effect, similar to the control sample (AGR).

A comparison of the release profiles of BG and GV also allows us to draw another conclusion about the release mechanism of these two APIs. The profiles of the curves (shape) for both APIs are very similar for the three hydrogels, AGR, Gel 1, and Gel 2. Both substances are hydrophilic, illustrating the release trends and the amount of released BG and GV in the series AGR > Gel 2 > Gel 1. Although BG has only two charged N atoms, which will affect the solubility of this substance in water, and GV has three N atoms, which allowed BG and GV to be used as salts, the shape of the release curves does not indicate that this parameter has a significant effect on the interaction of APIs with the solvent and their further release. However, when analyzing the release profiles of Figure 2B (BG) and Figure 2D (GV), differences in the release amount of these substances are observed. The percentage of released GV is about twice as high as BG for all the gels tested. It is probably related to the size of both molecules. Although the molar masses of both compounds are similar, BG (482.64 g mol^−1^) and GV (408.00 g mol^−1^), it should be noted that the size of the BG molecule is larger for BG due to the presence of ethyl substituents at the N atom. In GV, there are three methyl groups in the N atom. Considering all this, in the case of the amounts of BG and GV released from the gel, the dimensional considerations of both molecules are probably decisive in releasing these APIs from the hydrogels. To sum up, a higher amount of cooligomer in the hydrogel is needed to control the release of both hydrophilic GV and BG.

FUR, the only hydrophobic API, revealed interesting interactions with the hydrogel matrix (Figure 2C, Table S2). Except for the first 10 min of the test, Gel 1 and Gel 2 showed identical release patterns—very low but gradually increasing release of FUR. Interestingly, the control sample showed a similar pattern but with a much more pronounced total release. This indicates that, although AGR is mainly responsible for diffusion release, ([P(EMA)-*co*-(GMA)]-Xyl) acted as a hindering factor, regardless of its content, probably due to interactions between the hydrophobic part of the cooligomer ([P(EMA)-*co*-(GMA)]) and API. Adding the cooligomer was proven highly effective in limiting diffusion for poorly soluble and hydrophobic substances such as FUR.

When analyzing the release curves for all APIs, there may also be doubts about the total amount of released substance, such as BAC (Figure 2A), where the effect of molecular weight was very significant. Comparing BAC (Figure 2A), BG (Figure 2B), and FUR (Figure 2C), the amount of released API is about 5–6% in the study range (time), noting that in the case of FUR, the amount of released substance increases to about 10% in the last period studied. It should be emphasized, however, that many factors, as indicated above, affect the release mechanism of APIs, and we cannot compare their absolute release amounts, for which many of their parameters change simultaneously, i.e., size, solubility (API interactions with solvent), interaction with the polymer chain, etc. In the case of the indicated APIs, many of the indicated parameters change simultaneously; this applies not only to the tested APIs, which simultaneously affects the increase or inhibition of API release from the tested matrix, but in our studies, we also changed the composition of the tested hydrogels, in which at least the cross-linking of hydrogels was significantly changed. Therefore, we can formulate general conclusions regarding API release from the tested hydrogels by analyzing the above data. When comparing Gel 1 with Gel 2, it can be concluded that Gel 2 formulation allowed higher amounts of hydrophilic API to be released during the experiment than those in Gel 1, which is mostly pronounced in the case of BAC. In samples with BG or GV, an increased amount of API released was noticeable but not as significant as for BAC. The results of hydrophobic FUR suggest that smaller amounts of cooligomer should be used to control its release. Hydrogels, as substances containing substantial amounts of water in their composition, create more favorable conditions for hydrophilic substances to dissolve freely in the aqueous gel matrix.

### 2.6. Kinetic Models and Statistical Analysis of API Release Study

Modeling approaches contribute to understanding how different physicochemical properties of the polymer matrix influence the drug-release dynamics, enabling the optimization of formulations to achieve prolonged therapeutic effects. Therefore, several kinetic models (first-order [61], second-order [62], Baker–Lonsdale [63], Korsmeyer–Peppas [64]) were applied to fit the API release data from the [P(EMA)-*co*-(GMA)]-Xyl/AGR-based hydrogel (Supplementary Materials, Tables S3–S6). The zero-order kinetic model refers to the case when the dissolved form of the drug does not aggregate, the surface of the released matrix does not change, and equilibrium is not reached, reflecting the prolonged release process [61]. Second-order kinetics are characterized by a release rate that depends on the square of the concentration of the drug, often observed in gel formulations or systems where cross-linking influences release [62]. The Baker–Lonsdale kinetic model is recognized for controlled drug-release systems based on multiparticulate and polymer-based matrix [63]. The Korsmeyer–Peppas model is typically used to describe the general solute relationship behavior of controlled polymer matrices [64,65].

A curve-fitting analysis suggested that the kinetic models of the APIs release from Gels 1 and 2 conform to the Korsmeyer–Peppas model [64,66], which was fitted with the overall highest coefficients of determination (*R*^2^) for all studied samples, BAC, BG, GV, and FUR (Tables S3–S6). Therefore, cumulative API release in time is presented with the fitted Korsmeyer–Peppas kinetic model in Figure 2. The Korsmeyer–Peppas model is given below:

(1) $$\frac{Mt}{M_{\infty }}=K_{KP}\;t^{n}$$

where *Mt*/*M*_∞_ is the fraction of drug released (the relationship between the percentage of API released mass and the total added API to the hydrogel), *K_KP_* is the kinetic constant, which takes into account the properties of hydrogel and API, *t* is release time, and *n* is the diffusional release component indicative of drug release (Table 6). In the Supplementary Materials, the value of the released mass (*M*) was determined by the spectrophotometric method (Table S2). It was determined from the absorbance values of the solution at given time intervals and converted to API mass using the Lambert–Beer equation.

When *n* values are less than or equal to 0.45, the drug-release mechanism predominantly follows Fickian diffusion (Table 6) [59]. It means that the release is primarily governed by the diffusion of the drug through the polymeric matrix without significant involvement in polymeric chain relaxation or matrix erosion [67]. Comparing the values obtained for the Korsmeyer–Peppas kinetic model, all APIs were characterized with *n* parameters well below 0.45 for Gel 1 and Gel 2, with the *R*^2^ value equal to 0.9 or higher (Table S6). For Gel 1, the *n* values indicate a larger spread of values from 0.017 to 0.391, a higher content of cooligomer in the hydrogel, and for Gel 2, from 0.014 to 0.168 (values are given to three decimal places to illustrate the differences better). It should be noted, however, that the *R*^2^ values for Gel 1 are higher for all the APIs tested, which indicates the best fit of the Korsmeyer–Peppas kinetic model to the experimental data. For BG incorporated in Gel 1, the highest *n* value was calculated to equal 0.391, closer to 0.45 than for other APIs. It may suggest a transition toward the quasi-Fickian diffusion of BG from the hydrogel matrix (Table 6) [59,65], considering the small contribution of BG release mechanisms related to hydrogel relaxation. For Gel 2, the highest value of *n*, equal to 0.168, was calculated for FUR—the only hydrophobic substance tested. In Gel 2, we have twice as much (mass fraction) of AGR in the hydrogel as in Gel 1. It indicates the most inhibited diffusion of FUR in the hydrogel matrix and into the solution, which, compared to other APIs, may indicate some influence of the polymer relaxation process on the tested drug-release mechanism. Additionally, it should be noted that the same trend is observed for control samples (AGR). Also, in this case, the highest value of *n* equals 0.068, calculated for FUR. In this case, for all tested APIs, the values are an order of magnitude lower than for Gel 1 and Gel 2.

Understanding the *n* values aids in designing drug-delivery systems by allowing the tuning of polymer matrix properties—such as composition and cross-link density—to achieve desired drug-release profiles that can optimize therapeutic effects while minimizing side effects. In our studies, both Gel 1, a hydrogel with a higher amount of cooligomers in the matrix, and Gel 2 determined *n* values below 0.45, indicating a dominant diffusion-controlled release mechanism of the APIs from the hydrogels, which is desirable for applications requiring a consistent, controlled release of therapeutic agents.

The kinetic model also revealed (Table S6) that the kinetic constants (*K_KP_*) of Gel 2 were significantly higher than the ones for Gel 1 for hydrophilic APIs. It further supports the observations of the release profiles and proves the role of hydrophilic cooligomer ([P(EMA)-*co*-(GMA)]-Xyl) in changing the profile of API release from burst to more gradual. The opposite situation can be seen for hydrophobic FUR, where *K_KP_* of Gel 1 was higher than Gel 2, although the difference was much lower than for the other three APIs. Similar conclusions regarding differences between kinetic constants can be drawn from the analysis of second-order kinetic (Table S5). Interestingly, the Korsmeyer–Peppas predictions of a more extended release were not valid for Gel 1 for GV and BG (Table S6), where the data collected during the first six hours of the experiment suggested that the controlled and gradual release should continue. Still, the much faster achievement of a release equilibrium was observed. In the case of the release of BAC from Gel 1 and Gel 2 and FUR from the control matrix, the model predicted that the release equilibrium was reached; however, the experimental data showed a further gradual release of APIs. Overall, the API release behavior shows that traditional models cannot easily describe the obtained materials. The obtained drug-delivery systems based on ([P(EMA)-*co*-(GMA)]-Xyl) have complicated structural properties, including the heterogeneous structural nature of the formed hydrogel, which has hydrophobic and hydrophilic units in its structure as well as shorter cooligomer chains and longer AGR chains, which makes it impossible to precisely match the existing kinetic models of drug release from the hydrogel matrix and determine all parameters with high accuracy without more detailed analysis compromising different compositions of gels.

The LM model for continuous *y* was used to analyze data from API release studies, examining the relationship between % mass of API (~1), sample (control, Gel 1, Gel 2), time (time point), sample–time interaction (“sample × time”), and variability between individuals (ID as a random effect) (please see Supplementary Materials, Tables S7–S26) [68,69]. LMM was chosen because it accounts for fixed effects, representing population-level trends, and random effects, which capture variations between groups or individuals. The statistical analysis of API release studies using LMM is summarized in Tables S7–S26, and a summary of the main observations from the analysis is presented in Table 4, considering a post hoc comparison test for all samples.

The results for four API releases from Gel 1 and Gel 2 studies correlated with control indicated that the LMM used was appropriate for repeated measurements (e.g., the same samples at multiple time points) and allowed for taking into account differences between replicates and separate samples. The model fits the data well, as evidenced by the determination coefficients (*R*^2^), which are close to one for BAC and BG, while FUR and GV range from 0.817 to 0.864 (Table 4). These values for marginal *R*^2^ (the effect of sample and time) and conditional *R*^2^ (the differences between samples) can be explained by the fact that LMM accounts for all the variability [70]. Even with the lowest *R*^2^ value of 0.817, the statistical analysis suggests that approximately 81.7% of the variance in the dependent variable is accounted for by the LM model’s independent variables. This high value indicates a strong relationship between the independent and dependent variables, implying that LMM exhibits good predictive performance. The analyzed interactions in the data are reliable and statistically significant (*p* < 0.001) and indicate that the variables under this study significantly influence each other or the outcome being measured. In particular, the calculated “sample” effect (the differences between the control and Gel 1 and Gel 2), the calculated “time” effect (concentrations change over time—API is released), and the calculated sample–time interaction (the differences between the samples change over time) are statistically significant (*p* < 0.001). The calculated *p*-values for almost all analyzed data were equal to 0.001 with minor deviations, which nevertheless met the condition of a *p*-value less than 0.05 (Tables S9, S14, S19, and S24). For post hoc comparison tests, all sample *p*-values were adjusted using Tukey’s method and were *p*_Tukey_ < 0.001 (Tables S11, S16, S21, and S26).

The LM model fits BAC release well (Table 4 and Tables S7–S11), with a marginal *R*^2^ of 0.964 and a conditional *R*^2^ of 0.977. BAC release from Gel 1 and Gel 2 differed significantly from the control. The highest BAC release was observed for Gel 2, increasing to +6.75 mass units at the end of the experimental time interval (1440, 2880 min). The effect of BAC release is cumulative and significant for most time points—the effect of release increases with time.

The LM model fits BG release well (Table 4 and Tables S12–S16), with a marginal *R*^2^ of 0.930 and a conditional *R*^2^ of 0.959. Statistical analysis revealed differences in BG release for Gel 1 compared to the control. Gel 1 has significantly lower mass unit values than the control, on average by 2.6. The same trend was observed for Gel 2; the mass unit of BG released from the gel was, on average, 0.98 lower than the control. A gradual increase in BG release was observed for all matrices tested. For the sample–time interaction, the most significant effect was observed in the early hours—all early time points show substantial differences between samples.

The LM model fits FUR release quite well (Table 4 and Tables S17–S21), with a marginal *R*^2^ of 0.847 and a conditional *R*^2^ of 0.864. Gel 1 and Gel 2 released significantly less FUR than the control, with differences of approximately −4.6 and −4.8 mass units, respectively. Statistical analysis of the sample–time interaction shows that the differences between the matrices increase with time, with Gel 2 releasing more FUR than Gel 1. Furthermore, at the end of the study period (2880 min), Gel 1 released more FUR (−7.65 mass units) than Gel 2 (−6.99 mass units).

The LM model fits GV release well (Table 4 and Tables S22–S26), with a marginal *R*^2^ of 0.817 and a conditional *R*^2^ of 0.860. Statistical analysis indicates that GV release from Gel 1 is significantly and persistently reduced relative to control by approximately −4.0 mass units. In the case of Gel 2, that decrease is smaller but still statistically significant (−0.78 mass units). The GV–Gel 2 interaction is insignificant after 240 min, indicating a stabilization of the effect. However, for Gel 1, the %mass release of GV increases, indicating a strong GV–Gel 1 interaction effect in the examined time window.

**Table 4:** General conclusions from the statistical analysis of API release studies using LMM.

API	*R* ^2^		The Strongest Effect	GEL 1 vs. GEL 2	Comment
	Marginal *	Conditional **			
BAC	0.964	0.977	Gel 2 > Gel 1 > control	Yes	High, rising release
BG	0.930	0.959	Gel 1 < Gel 2 < control	Yes	Gel 1 inhibits more strongly than Gel 2
FUR	0.847	0.864	Gel 1 ≈ Gel 2 < control	Slightly	Long-lasting effect of Gels 1 and 2
GV	0.817	0.860	Gel 1 < Gel 2 < control	Yes	Gel 1 durable and strong, Gel 2 weaker

* Marginal *R*^2^ (explains the effect of sample and time); ** conditional *R*^2^ (explains the differences between samples together).

The post hoc comparison test of samples using Tukey’s method shows that the model is statistically significant (*p* < 0.001) and can lead to conclusions that the differences in API release between different gel formulations were noticed (Table 4, Table S11 for BAC, Table S16 for BG, Table S21 for FUR, and Table S26 for GV). The Gel 1 and Gel 2 formulations demonstrate statistically significant differences in release profiles, as identified through a post hoc comparison test, in the hydrophilic and hydrophobic APIs from the matrix. Gels 1 and 2 have a hydrophilic character, and a prolonged release of APIs was observed for both gels, regardless of the hydrophilic–hydrophobic nature of the APIs. It should be noted, however, that the more hydrophilic nature of Gel 1 (higher cooligomer content in the matrix) inhibits the release of hydrophilic drugs (API: BAC, BG, and GV). Analyzing the post hoc comparison test, it can be concluded that if a faster release of a hydrophilic drug is desired, the Gel 2 formulation should be used. In contrast, the Gel 1 formulation should be used if a prolonged release profile is necessary. The release was consistent from Gel 1 and Gel 2 only for hydrophobic FUR (Table 4), which means that in this case, no effect of the gel formulation on the release profile of FUR from the matrix was observed. In case of hydrophobic drugs, both gel formulations can be used for therapies, where a consistent release of drugs is desired, because the drug release can positively contribute to treatment. Thus, our findings from post hoc comparison tests may aid in optimizing gel formulations consisting of hydrophilic cooligomer ([P(EMA)-*co*-(GMA)]-Xyl) in an AGR matrix that achieve desired pharmacological outcomes.

## 3. Materials and Methods

### 3.1. Materials

1,8-diazabicyclo(5.4.0)undec-7-ene (DBU, 98%), agarose (AGR, low EEO), azobisisobutyronitrile (AIBN, ≥95%), bacitracin (BAC) (from *Bacillus licheniformis*, ≥65 IU/mg), brilliant green (BG, dye content~90%, powder), dabsyl chloride (≥97.5%), ethyl methacrylate (EMA, ≥99%, (stabilised) for synthesis), ethyl acetate (ACS grade, ≥99.5%), glycidyl methacrylate (GMA, ≥97%), gentian violet (GV, United States Pharmacopeia (USP) Reference Standard), phosphate buffered saline (PBS, pH = 7.4, powder), sodium lauryl sulfate (SLS, Pharmaceutical Secondary Standard; Certified Reference Material), xylitol (Xyl, ≥99%) and diiodomethane (99%, liquid) were purchased from MERCK (Poznań, Poland). Acetone, dimethylformamide (DMF), glycerol, ethanol, heptane, and hexane were purchased from POCH (Gliwice, Poland), LB Broth with agar (Lennox) from BioShop (EPRO, Warsaw, Poland), furazidine (FUR) from Biosynth (Bratislava, Slovakia), sodium bicarbonate from Chempur (Piekary Śląskie, Poland), and sodium carbonate from Standard (Lublin, Poland). All reagents were of analytical grade and used without prior purification.

### 3.2. Methods

#### 3.2.1. Drug-Loaded [P(EMA)-*co*-(GMA)]-Xyl Gel Preparation

To prepare hydrogel matrices, two different mass ratios of oligomer ([P(EMA)-*co*-(GMA)]-Xyl) to AGR were used, as presented in Table 5. Gel 1 with [P(EMA)-*co*-(GMA)]-Xyl to AGR ratio of 2:1, and Gel 2 with [P(EMA)-*co*-(GMA)]-Xyl to AGR ratio of 1:1. If not indicated, the Gel 2 formulation was taken for further testing. BAC (11.5 mg for Gel 1, 10.78 mg for Gel 2), BG (7.5 mg for Gel 1, 7 mg for Gel 2), FUR (7.5 mg for Gel 1, 7 mg for Gel 2), and GV (3.75 mg for Gel 1, 3.5 mg for Gel 2) were mixed (each separately) with [P(EMA)-*co*-(GMA)]-Xyl (100 mg for Gel 1, 50 mg for Gel 2) and transferred to a plastic tube (diameter 0.4 cm) sealed at the end. A 10% AGR solution in PBS (500 µL, PBS 0.01 M, pH = 7.4) was carefully injected into this tube and mixed until the substance was uniformly dispersed, giving [P(EMA)-*co*-(GMA)]-Xyl/AGR (Table 5).

**Table 5:** [P(EMA)-*co*-(GMA)]-Xyl-based gel formulation for API release and microbiological studies.

AGR (mg)	PBS (mL)	[P(EMA)-*co*-(GMA)]-Xyl (mg)		API				
				Conc. (%)	BAC (mg)	BG (mg)	FUR (mg)	GV (mg)
50.0	0.45	50.0 Gel 2 0.00 Control	Microbiological studies	0.1	10.1	10.1	10.1	10.1
				0.5	50.5	50.5	50.5	50.5
				1.0	101.0	101.0	101.0	101.0
				2.0	202.0	202.0	202.0	202.0
		100.0 Gel 1	Release studies	-	11.5	7.5	7.5	3.75
		50.0 Gel 2		-	10.78	7.0	7.0	3.5
		0.00 Control						

**Figure 3:**
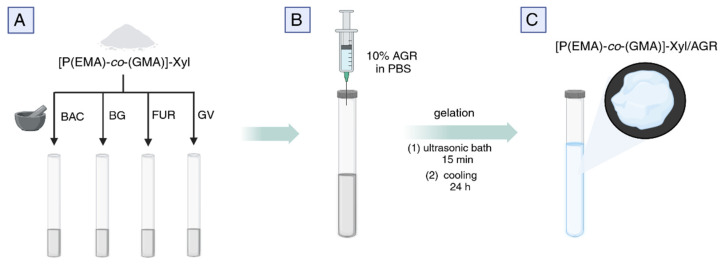
Schematic representation of drug-loaded [P(EMA)-*co*-(GMA)]-Xyl/AGR gel preparation. (**A**) Preparation of [P(EMA)-co-(GMA)]-Xyl oligomers with four APIs: BAC, BG, FUR, and GV. (**B**) Adding 10% AGR solution in PBS to all prepared API cooligomers and subjecting them ultrasonic mixing for 15 min. The gelation process takes place within 24 h at a reduced temperature. (**C**). Schematic image of the formed gel.

Control samples were prepared similarly, except that no [P(EMA)-*co*-(GMA)]-Xyl oligomer was used. The plastic tubes were put in an ultrasonic bath for 15 min to homogenize the mixture and then placed in the refrigerator overnight to gel, giving [P(EMA)-*co*-(GMA)]-Xyl/AGR gel. Thus prepared, the tubes were ready to determine the release of the API. The procedure for obtaining drug-loaded gels is shown in Figure 3.

#### 3.2.2. Antibacterial Properties of [P(EMA)-*co*-(GMA)]-Xyl-Based Gel

[P(EMA)-*co*-(GMA)]-Xyl/AGR gel with various antibiotic content (Table 5) were prepared to assess drug release and their influence on bacterial growth in a solid medium. Briefly, 20 µL of each formulated Gel 2 was applied to autoclaved blotting paper disks (diameter 5.5 mm) and left for 30 min at 4 °C. Next, disks were placed on LB AGR (Lennox) dishes with freshly spread bacteria strains, including *Escherichia coli* DH5α, *Pseudomonas aeruginosa* ATCC 9027, or *Staphylococcus epidermidis* ATCC 12228 and left for 30 min at 21 °C. Finally, dishes were incubated at 37 °C for 18 h. After that, bacteria growth inhibition zones were analyzed and measured. Gel disks without any antibiotics were used as a control. As a second control, 0.5% AGR gel applied on blotting paper disks (without [P(EMA)-*co*-(GMA)]-Xyl added) was prepared without or with the same antibiotic contents, similarly to the [P(EMA)-*co*-(GMA)]-Xyl-based gel. The experiments were performed in duplicates.

#### 3.2.3. Contact Angle

Contact angle (CA) examination was conducted using the sessile drop method and the contact-angle goniometer Rame-Hart 90-U3-PRO (ramé-hart instrument co, Succasunna, NJ, USA). Before the examination, a solution of 50 mg [P(EMA)-*co*-(GMA)]-Xyl dissolved in ethyl acetate was applied to the glass plate and left for the solvent to evaporate. Glass plates were then placed on the goniometer table and leveled. The droplet of measuring liquid (water, diiodomethane, or glycerol) was then placed on top of the samples’ surfaces with a micro syringe (Hamilton Company, Bonaduz, Switzerland), and measurements were conducted. Drop Image Pro software (https://www.ramehart.com/dropimage_pro.htm, accessed on 28 June 2025) was then used to calculate the contact angle and surface free energy (SFE) results using the Good–van Oss–Chaudhury model. CA was given as an average from three different measurements.

#### 3.2.4. Rheological Studies of Gel

Rheological properties were analyzed using Anton Paar Physica MCR 301 rheometer (Anton Paar GmbH, Graz, Austria). Two types of measurements were performed: temperature and frequency sweep. Samples had the same ratio between the oligomer and AGR as Gel 1, but a different amount of solvent was used (150 mg oligomer, 75 mg AGR, 0.75 mL water). After mixing the substrates, the sample was placed in the center of a heating plate. The sample was then heated to 37 °C for 2 min and pressed until the measurement gap, equal to 1.5 mm, was reached.

The temperature sweep test aimed to determine the gel-to-sol transition temperature. For this purpose, the sample was cooled to 5 °C and thermostated for 20 min. The elastic response was observed under the following parameters: strain: 1%; frequency: 10 Hz; temperature range: 5–70 °C; temperature increase rate: 1 °C/min.

Frequency sweep, on the other hand, was conducted to determine the shear frequency at which the gel-to-sol transition occurs. For this purpose, the sample was thermostated at 37 °C for 20 min and then tested under the following conditions: strain: 2.5%; frequency range: 10^−3^–10^2^ Hz; logarithmic increment; 26 measurement points; testing rate: 1 point per minute.

#### 3.2.5. BG Degradation Studies

To analyze the possible decomposition and its cause, eight solutions were prepared with 5 mg of dissolved BG: four with water and four with PBS. The solutions were stored for 48 h in different conditions, including light (daylight/blackout) and temperature (23 °C/37 °C) exposition. Then, the degradation susceptibility of the BG was studied by comparing the BG content with the BG water solution prepared ex tempore.

#### 3.2.6. In Vitro Release Studies of APIs

All release studies were performed based on the Pharmacopeia release method with modifications [71]. The analysis was carried out continuously for 48 h. There were defined time points at which the solution was sampled for spectral analysis, and the deficiency was refilled with a pure medium at the same temperature. Tests were performed in the range of 1 to 2880 min. PBS was used to release FUR and GV, whereas carbonate buffer was used for BAC. Pure water was applied to the BG release study.

The BAC determination was carried out as follows. Carbonate buffer (30 mL, 0.05 M, pH = 9) was added to a beaker, placed on a magnetic stirrer (100 rpm), and heated to 37 °C, being protected from evaporation. When the set temperature was reached, the plastic tube filled with [P(EMA)-*co*-(GMA)]-Xyl/AGR (or control sample—AGR) tip was cut off and dipped into the solution. A sample (100 µL) was collected at specified intervals, and the deficit was replaced with a fresh aliquot of carbonate buffer. Then, 1 mL of dabsyl chloride solution (1 × 10^−3^ mol dm^−3^) was added to the sample and placed in a water bath at 70 °C for 15 min. After this time, the sample was left to cool for 15 min. The sample was then filled with acetone to a volume of 5 mL. Absorbance was measured using a spectrophotometer (Spekol 1300, Analytik Jena GmbH, Jena, Germany) at 474 nm.

GV were determined as follows. PBS (30 mL, 0.01 M, pH = 7.4) was poured into a beaker, placed on a magnetic stirrer (100 rpm), and heated to 37 °C while protecting it from evaporation. When the temperature was reached, the plastic tube with [P(EMA)-*co*-(GMA)]-Xyl/AGR (or control sample—AGR) tip was cut off and dipped into the solution. A sample (100 µL) was taken at defined intervals, and the deficiency was refilled with a fresh portion of PBS. Spectrophotometric analysis was carried out at a wavelength of 586 nm.

BG was determined using a method similar to the determination of GV. Due to the faster decomposition of this substance in PBS than in water, the previous method was modified by replacing the PBS with MiliQ water (Milli-Q® IQ 7000 Ultrapure Water Purification System, MERCK, Warszawa, Poland). The other parameters of the procedure remained unchanged. Absorbance was measured at a wavelength of 625 nm.

FUR was determined using a similar method with some modifications. Briefly, 0.6 g of SLS was added to a PBS solution (30 mL, 0.01 mol dm^−3^, pH = 7.4) to obtain 2% solution. Absorbance was measured at a wavelength of 292 nm.

#### 3.2.7. Kinetic Models

All considerable data for API release studies are collected and presented in Table S2. The models were chosen to analyze the API release mechanism to fit the experimental data using OriginPro 9.0 software (OriginLab Corporation, Northampton, MA, USA). The obtained release values were fitted using the several models described in the Supplementary Materials. For the consideration of drug release, the drug-release profile was fitted in the Korsmeyer–Peppas model [64,65,72]. The kinetic parameters were calculated using Equation (1) for BAC, GV, BG, and FUR for cylindrical-shaped matrices [59,65]. The *n* value is used to characterize different release mechanisms, as given in Table 6. Additional descriptions of the studies are included in the Supplementary Materials.

**Table 6:** Diffusion exponent and solute release mechanism for cylindrical shape diffusion [59,65].

Exponent (*n*)	Overall Solute Diffusion Mechanism
≤0.45 (0.43)	Fickian diffusion
0.45 (0.43) < *n* < 0.89 (0.85)	Anomalous (non-Fickian) diffusion
0.89 (0.85) < *n* < 1	Case-II transport
*n* > 1	Super case II transport

Several kinetic models were chosen to analyze the API release kinetic parameters of gels to fit the experimental data (Baker–Lonsdale, first-order, second-order and Korsmeyer–Peppas models), using OriginPro 9.0 software (OriginLab Corporation, Northampton, MA, USA). API release kinetic parameters of gels for different mathematical models were calculated using only average values of API release (%*M_eq_*) in the function of time (*t*) for each sample (*n* = 3). The Baker–Lonsdale model was developed with the consideration of spherical matrices (Equation (2)) [73]:

(2) $$K_{BL}t=\frac{3}{2}\left(1-{\left(1-\frac{M_{t}}{M_{eq}}\right)}^{\frac{2}{3}}\right)-\frac{M_{t}}{M_{e}}$$

where *M_t_* is the API release at a time interval (*t*) (%), *M_eq_* is equilibrium API release (%) and *K_BL_* is the Baker–Lonsdale diffusion kinetic constant (min^−1^). The Baker–Lonsdale model applies to cylindrical matrices where drug release occurs primarily through diffusion [63]. In the case of first-order kinetic, the API release value at any time (*t*) is proportional to the uptake of the API release before reaching equilibrium API release (*M_eq_*) [54]. The first-order kinetic is represented by Equation (3):

(3) $$\frac{dM}{dt}=K_{1}(M_{eq}-M_{t})$$

where *M_eq_* is equilibrium API release (%), *M_t_* is API release at a time interval (*t*) (%) and *K*_1_ is the first order API release kinetic constant (min^−1^). First-order kinetics describe a situation where the rate of drug release is proportional to the remaining concentration of the drug in the formulation [61]. Equation (3) can be integrated to the nonlinear Equation (4):

(4) $$M=M_{eq}(1-e^{-K_{1}t})$$

API release parameters were also calculated according to the second-order kinetic using Equation (5) [74]:

(5) $$\frac{dM}{dt}=K_{2}{\left(M_{eq}-M_{t}\right)}^{2}$$

where *M_eq_* is equilibrium of API release (%), *M_t_* is API release at a set time (*t*) (%) and *K*_2_ is the second-order API release kinetic constant (min^−1^). Second-order kinetics are characterized by a release rate that depends on the square of the concentration of the drug, often observed in gel formulations or systems where cross-linking influences release [62]. Equation (5) integrates to the nonlinear Equation (6):

(6) $$M=\frac{K{M_{eq}}^{2}t}{1+KM_{eq}t}$$

with the assumption that *M*_0_ = *M* = 0 and *t*_0_ = *t* = 0. The Korsmeyer–Peppas nonlinear diffusion model was defined using Equation (1) [72]:

(7) $$\frac{Mt}{M_{\infty }}=K_{KP}\;t^{n}$$

where *Mt/M*_∞_ is the fraction of drug released (the relationship between the percentage of API released mass and the total added API to the hydrogel), *K_KP_* is the kinetic constant, which takes into account the properties of hydrogel and API, *t* is the release time, and *n* is the diffusional release component indicative of drug release (Table 6).

#### 3.2.8. Statistical Analysis

Data obtained from antibacterial studies were analyzed using one-way ANOVA and Tukey’s honest significant difference post hoc comparison test (*p* < 0.05) using Origin Pro 9.0 software (OriginLab Corporation, USA).

API release data were analyzed using LMM with fixed effects for sample type, time, and their interaction and a random intercept for sample ID. Data were analyzed using LMM for continuous *y*, normal distribution of residuals, and dependent variable scores. Degrees of freedom were estimated via the Satterthwaite method. The post hoc comparison test was adjusted using Tukey’s honest significant difference using JAMOVI (https://www.jamovi.org/, accessed on 28 June 2025).

## 4. Conclusions

In summary, our study shows the possibility of using the [P(EMA)-*co*-(GMA)]-Xyl-based gels to release various therapeutic substances with antibacterial properties. Gel 2 revealed similar or higher antimicrobial properties than the control gel (AGR). For FUR-*E. coli* (slightly) and GV-*E. coli* (significantly), BG-*S. epidermidis* and BG-*P. aeruginosa* (slightly) pairs, a more potent inhibition of bacterial growth was observed for Gel 2. Finally, no or modest antimicrobial effects were observed for the tested gels and controls for the following pairs: BAC-*E. coli*, BAC-*P. aeruginosa*, and FUR-*P. aeruginosa*. The hydrogels with the APIs efficiently inhibit bacteria growth with low doses of drugs, and our findings are statistically significant, confirmed with ANOVA analysis at *p* = 0.05. Bacteria inhibition zones were observed with even small API concentrations, which indicates the possibility of using low doses of antibiotics to maintain the antimicrobial activity of the modified gels.

The higher cooligomer content in Gel 1 leads to a more hydrophilic matrix character, resulting in a slower and more controlled API release due to matrix-API interaction. It is particularly evident in substances of different polarities, such as hydrophilic: BAC, BG, and GV, and hydrophobic FUR. The smaller amount of gel’s cooligomer changes its structure to a denser one, translating into a more difficult diffusion of the substances and their lower API release values for the substances with higher molar mass value. A pronounced burst-release effect in the initial phase accompanies it. The prolonged release of APIs characterizes hydrogels—the amount of APIs released in a very short time (a few minutes) is approximately 10%. However, the final experimental values of the mass of APIs (calculated to the concentration in 1 mL of acceptor fluid) significantly exceed the minimum inhibitory concentration of APIs. Therefore, the tested gels have a pharmacological effect even at a low released API concentration. Comparing the calculated values obtained for the Korsmeyer–Peppas kinetic model, all APIs were characterized with *n* parameters well below 0.45 for Gel 1 and Gel 2, with the *R*^2^ value equal to 0.9 or higher, confirming that the drug-release mechanism from [P(EMA)-*co*-(GMA)]-Xyl/AGR gel predominantly follows Fickian diffusion.

LMM was used to analyze data from API release studies, examining the relationship between % mass of API, sample, time, sample–time interaction, and variability between individuals. The LM model fits the data well, as evidenced by *R*^2^, the analyzed interactions in the data are reliable and statistically significant (*p* < 0.001), and indicate that the variables under this study significantly influence each other or the outcome being measured. The post hoc comparison test of samples using Tukey’s method shows that the model is statistically significant (*p* < 0.001) allows us to draw the conclusion that differences in API release between different gel formulations were noticed. The Gel 1 and Gel 2 formulations demonstrate statistically significant differences in release profiles in the hydrophilic and hydrophobic APIs from the matrix. For Gels 1 and 2, a prolonged release of APIs was observed. It should be noted, however, that the more hydrophilic nature of Gel 1 inhibits the release of hydrophilic drugs (BAC, BG, and GV). The release was consistent for hydrophobic FUR from Gel 1 and Gel 2.

The results of the antibacterial substances release study confirmed that the cooligomers are an effective matrix for an extended drug-release system. Overall, the materials presented in this study could be used to produce dressings with antimicrobial activity for wound treatment. Further studies involving [P(EMA)-*co*-(GMA)]-Xyl/AGR gel should include cytotoxicity analysis, together with the assessment of possible combinations with other materials to obtain a complete dressing. Further studies on cell interactions, compatibility with different materials, susceptibility to sterilization, and long-term shelf life should be conducted to confirm this.

## Figures and Tables

**Table 2:** Contact-angle results.

	Mean Contact Angle (°)	Standard Deviation (°)
Water	60.74	0.12
Diiodomethane	18.46	0.08
Glycerol	61.24	0.10

**Table 3:** The effect of different conditions of temperature and light availability on the concentration of BG in water or PBS as a medium. Absorbance was measured after 48 h relative to a control sample prepared just before spectrophotometric measurement.

Sample	Medium		Temperature		Light	Concentration
	Water	PBS	23 °C	37 °C		(mmol dm^−3^)
1	+	-	+	-	Daylight	0.063
2	-	+	+	-	Daylight	0.019
3	+	-	-	+	Daylight	0.069
4	-	+	-	+	Daylight	0.008
5	+	-	+	-	Blackout	0.058
6	-	+	+	-	Blackout	0.037
7	+	-	-	+	Blackout	0.070
8	-	+	-	+	Blackout	0.020
Control	+	-	prepared ex tempore			0.071

## Data Availability

The data supporting this article have been included in the manuscript and as part of the ESI.

## Supplementary Materials

The following supporting information can be downloaded at: https://www.mdpi.com/article/10.3390/molecules30153083/s1, Figure S1: Contact angle measurements; Figure S2: Storage modulus (G’) and loss modulus (G”) obtained for sample Gel 1 from temperature sweep (left) and frequency sweep (right) measurements; Table S1: Summary of rheological measurements obtained for sample Gel 1 from temperature and frequency sweep; Table S2: Results of spectrophotometric measurements of [P(EMA)-*co*-(GMA)]-Xyl/AGR (Gel 1 and Gel 2) loaded with BAC, BG, FUR and GV, respectively, presented as a percentage of the mass released (M) relative to the initial API quantity; Table S3: API release kinetic parameters of gels calculated using Baker-Lonsdale kinetic model; Table S4: API release kinetic parameters of gels calculated using first order kinetic model; Table S5: API release kinetic parameters of gels calculated using second order kinetic model; Table S6: API release kinetic parameters of gels calculated using Korsmeyer–Peppas kinetic model; Table S7: BAC release LMM fit; Table S8: BAC release—Fixed Effects Omnibus Tests; Table S9: BAC release—Parameter Estimates (Fixed coefficients); Table S10: BAC release—Random components; Table S11: BAC release—Post hoc comparison: SAMPLE; Table S12: BG release LMM fit; Table S13: BG release—Fixed Effects Omnibus Tests; Table S14: BG release—Parameter Estimates (Fixed coefficients); Table S15: BG release—Random components; Table S16: BG release—Post hoc comparison: SAMPLE; Table S17: FUR release LMM fit; Table S18: FUR release—Fixed Effects Omnibus Tests; Table S19: FUR release—Parameter Estimates (Fixed coefficients); Table S20: FUR release—Random components; Table S21: FUR release—Post hoc comparison: SAMPLE; Table S22: GV release LMM fit; Table S23: GV release—Fixed Effects Omnibus Tests; Table S24: GV release—Parameter Estimates (Fixed coefficients); Table S25: GV release—Random components; Table S26: GV release—Post hoc comparison: SAMPLE.
