# Supplementary material for: Formation and Characterization of Xylitol-Modified Glycidyl Methacrylate-co-Ethyl Methacrylate Matrices for Controlled Release of Antimicrobial Compounds
Source: Molecules. 2025 Jul 23;30(15):3083. doi: 10.3390/molecules30153083 (PMC12348667; doi:10.3390/molecules30153083)
Supplement: Supplementary file 1 [file molecules-30-03083-s001.zip › molecules-3757561-supplementary.pdf]

Supplementary Materials

Formation and Characterization of Xylitol-Modified Glycidyl Methacrylate-*co*-Ethyl Methacrylate Matrices for Controlled Release of Antimicrobial Compounds

Adam Chyzy[1], Przemysław Gnatowski[2,3], Edyta Piłat[3], Maciej Sienkiewicz[3], Katarzyna Wozniak[4], Marta Wojnicka[4], Krzysztof Brzezinski[4*], Marta E. Plonska-Brzezinska[1*]

[1] Department of Organic Chemistry, Faculty of Medicine with the Division of Dentistry and Division of Medical Education in English, Medical University of Bialystok, Mickiewicza 2A, 15-222 Bialystok, Poland; adam.chyzy@sd.umb.edu.pl; marta.plonska-brzezinska@umb.edu.pl,

[2] Department of Environmental Toxicology, Faculty of Health Sciences, Medical University of Gdańsk, Dębowa 23A, 80-204 Gdańsk, Poland, przemyslaw.gnatowski@gumed.edu.pl,

[3] Department of Polymer Technology, Faculty of Chemistry, Gdansk University of Technology, Narutowicza St. 11/12, 80-233 Gdansk, Poland, przemyslaw.gnatowski@pg.edu.pl, edyta.pilat@pg.edu.pl, maciej.sienkiewicz@pg.edu.pl,

[4] Department of Structural Biology of Prokaryotic Organisms, Institute of Bioorganic Chemistry, Polish Academy of Sciences, Noskowskiego 12/14, Poznan, 61-704, Poland; kawozniak@ibch.poznan.pl, mwojnicka@ibch.poznan.pl, kbrzezinski@ibch.poznan.pl,

* Correspondence: marta.plonska-brzezinska@umb.edu.pl (M.E.P.-B.)

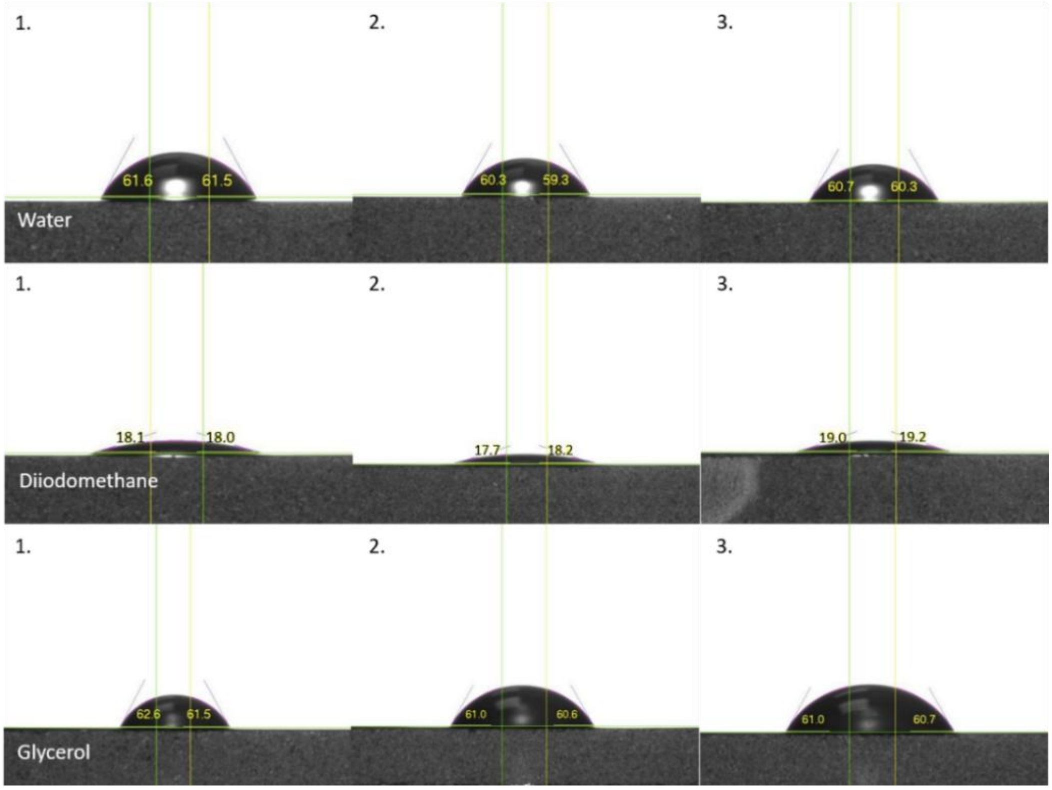

Figure S1. Contact angle measurements. Numbers represent the order in which test were taken.

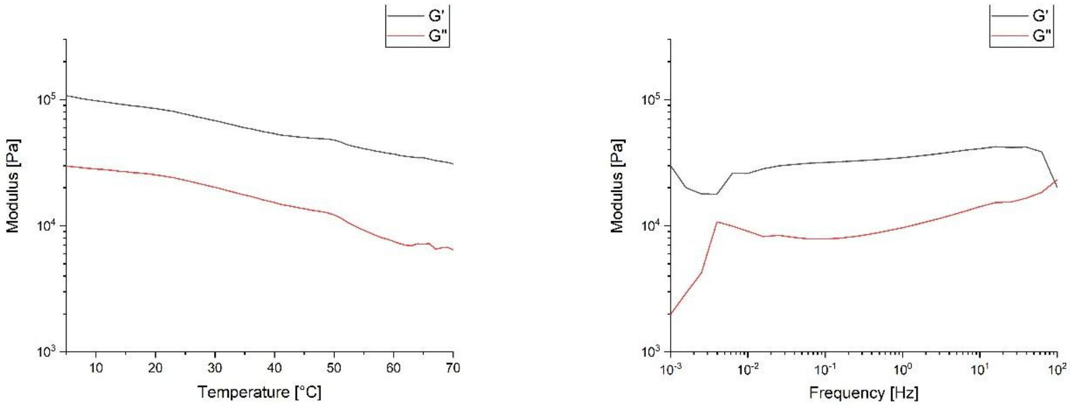

Figure S2. Storage modulus (G′) and loss modulus (G″) obtained for sample Gel 1 from temperature sweep (left) and frequency sweep (right) measurements.

Table S1. Summary of rheological measurements obtained for sample Gel 1 from temperature and frequency sweep.

Parameter	Temperature sweep			Frequency sweep				
	**5 °C**	**20 °C**	**37 °C**	**0.01 Hz**	**0.1 Hz**	**1 Hz**	**10 Hz**	**100 Hz**
G′ [kPa]	107.6	85.0	57.4	29.9	26.0	31.7	40.9	20.1
G″ [kPa]	29.7	25.3	16.6	1.9	9.1	7.8	14.2	23.1
Tanδ [1]	0.276	0.298	0.289	0.066	0.348	0.247	0.346	1.153

Several kinetic models were chosen to analyse the API release kinetic parameters of gels to fit the experimental data (Baker-Lonsdale, first order, second order and Korsmeyer-Peppas models), using OriginPro 9.0 software (OriginLab Corporation, Northampton, USA). API release kinetic parameters of gels for different mathematical models were calculated using only average values of API release (%M_{eq}) in the function of time (t) for each sample (n=3).
The Baker-Lonsdale model was developed with the consideration of spherical matrices (Eq. (S1)) [1]:

$$K_{BL}t = \frac{3}{2}\left(1 - \left(1 - \frac{M_t}{M_{eq}}\right)^{\frac{2}{3}}\right) - \frac{M_t}{M_e} \quad \text{(S1)}$$

where M_t is API release at a time interval (t) (%), M_{eq} is equilibrium API release (%) and K_{BL} is the Baker-Lonsdale diffusion kinetic constant (min^{-1}). The Baker-Lonsdale model applies to cylindrical matrices where drug release occurs primarily through diffusion [2], [3].
In the case of first order kinetic, the API release value at any time (t) is proportional to the uptake of the API release before reaching equilibrium API release (R_{eq}) [4]. The first order kinetic is represented by Equation (S2):

$$\frac{dM}{dt} = K_1(M_{eq} - M_t) \quad \text{(S2)}$$

where M_{eq} is equilibrium API release (%), M_t is API relase at a time interval (t) (%) and K_1 is the first order API release kinetic constant (min^{-1}). First-order kinetics describe a situation where the rate of drug release is proportional to the remaining concentration of the drug in the formulation [4]. Equation (S2) can be integrated to the nonlinear Equation (S3):

$$M = M_{eq}(1 - e^{-K_1 t}) \quad \text{(S3)}$$

API release parameters were also calculated according to the second order kinetic using the Equation (S4) [5]:

$$\frac{dM}{dt} = K_2\,(M_{eq} - M_t)^2 \quad \text{(S4)}$$

where: M_{eq} is equilibrium of API release (%), M_t is API release at a set time (t) (%) and K_2 is the second order API release kinetic constant (min^{-1}). Second-order kinetics are characterized by a release rate that depends on the square of the concentration of the drug, often observed in gel formulations or systems where cross-linking influences release [5], [6]. Equation (S4) integrates to the nonlinear Equation (S5):

$$M = \frac{K{M_{eq}}^2 t}{1 + KM_{eq}t} \quad \text{(S5)}$$

with the assumption that $M_0 = M = 0$ and $t_0 = t = 0$.
Korsmeyer-Peppas nonlinear diffusion model can be defined using Equation (S6) [7]:

$$M = K_{KP}\,t^n \quad \text{(S6)}$$

where M is API release (%), K_{KP} is API release rate constant (min^{-1}), t represents the set time interval (min), and n is diffusion exponent.

Table S2. Results of spectrophotometric measurements of [P(EMA)-*co*-(GMA)]-Xyl/AGR (Gel 1 and Gel 2) loaded with BAC, BG, FUR and GV, respectively, presented as a percentage of the mass released (*M*) relative to the initial API quantity. SD (Standard Deviation).

Time [min]	M_{BAC} [%]						M_{BG} [%]					
	**Control**	**SD**	**Gel 1**	**SD**	**Gel 2**	**SD**	**Control**	**SD**	**Gel 1**	**SD**	**Gel 2**	**SD**
0	0.00	0.00	0.00	0.00	0.00	0.00	0.00	0.00	0.00	0.00	0.00	0.00
1	5.34	0.58	6.41	0.20	9.79	0.34	5.05	0.09	0.28	0.06	2.00	1.20
2	5.56	0.87	6.44	0.20	9.83	0.31	5.13	0.01	0.64	0.18	2.81	1.34
3	5.65	0.74	6.50	0.15	9.90	0.30	5.13	0.01	0.81	0.24	3.14	1.27
4	5.71	0.76	6.56	0.12	9.98	0.27	5.14	0.01	1.00	0.32	3.47	1.18
5	5.66	0.82	6.61	0.12	10.04	0.27	5.15	0.02	1.05	0.28	3.83	1.04
10	5.72	0.74	6.64	0.14	10.11	0.27	5.17	0.02	1.53	0.31	4.19	0.51
15	5.84	0.66	6.65	0.15	10.16	0.26	5.17	0.02	1.81	0.37	4.36	0.34
20	5.72	0.50	6.66	0.12	10.18	0.27	5.18	0.03	2.00	0.37	4.41	0.33
25	5.82	0.51	6.68	0.12	10.21	0.25	5.19	0.03	2.18	0.37	4.42	0.33
30	5.81	0.44	6.71	0.09	10.25	0.24	5.31	0.21	2.24	0.40	4.44	0.34
45	5.73	0.49	6.71	0.10	10.26	0.26	5.32	0.22	2.42	0.31	4.55	0.39
60	5.69	0.53	6.74	0.09	10.29	0.24	5.34	0.20	2.48	0.34	4.59	0.44
90	5.74	0.45	6.76	0.10	10.33	0.24	5.35	0.20	2.86	0.42	4.62	0.46
120	5.72	0.69	6.81	0.12	10.39	0.27	5.35	0.19	3.07	0.28	4.63	0.48
150	5.79	0.63	6.84	0.09	10.39	0.22	5.43	0.17	3.46	0.47	4.65	0.48
180	5.83	0.68	6.89	0.11	10.48	0.24	5.46	0.17	3.57	0.46	4.67	0.46
210	5.75	0.73	6.94	0.12	10.53	0.26	5.56	0.03	3.75	0.55	4.70	0.45
240	5.76	0.66	6.99	0.12	10.58	0.28	5.57	0.02	3.86	0.59	4.84	0.33
270	5.61	0.68	7.04	0.14	10.65	0.31	5.57	0.02	3.91	0.53	4.87	0.32
300	5.64	0.77	7.09	0.14	10.69	0.34	5.57	0.02	3.96	0.55	4.90	0.29
330	5.88	0.82	7.15	0.14	10.77	0.34	5.59	0.03	4.03	0.57	4.90	0.29
360	5.79	0.84	7.24	0.13	10.85	0.34	5.59	0.02	4.09	0.58	4.95	0.26
1440	6.07	0.75	8.82	0.76	12.42	1.29	5.63	0.05	4.27	0.44	5.00	0.33
2880	6.09	0.94	9.59	1.12	12.84	1.23	5.65	0.05	4.40	0.33	5.24	0.16

Time [min]	M_{FUR} [%]						M_{GV} [%]					
	**Control**	**SD**	**Gel 1**	**SD**	**Gel 2**	**SD**	**Control**	**SD**	**Gel 1**	**SD**	**Gel 2**	**SD**
0	0.00	0.00	0.00	0.00	0.00	0.00	0.00	0.00	0.00	0.00	0.00	0.00
1	2.25	2.11	1.55	0.58	1.04	0.81	8.77	1.56	1.68	0.16	6.10	3.26
2	3.79	2.22	1.47	0.35	1.12	0.87	8.93	1.47	1.92	0.32	6.87	2.86
3	5.65	1.34	1.73	0.92	1.22	0.86	9.08	1.51	2.39	0.09	7.67	2.36
4	6.21	0.85	1.76	0.72	1.22	0.80	9.18	1.48	2.86	0.26	7.97	2.33
5	6.40	0.98	1.76	0.79	1.22	0.81	9.38	1.40	3.26	0.45	8.20	2.55
10	6.47	1.00	1.78	0.79	1.43	1.00	9.64	1.29	3.70	0.70	8.50	2.31
15	6.51	1.04	1.79	0.81	1.52	1.06	9.72	1.28	4.23	0.60	8.66	2.08
20	6.71	1.12	1.86	0.87	1.62	1.14	9.79	1.29	4.32	0.67	8.79	1.91
25	6.81	1.17	1.87	0.84	1.67	1.14	9.84	1.30	4.84	0.57	8.80	1.91
30	6.92	1.19	1.97	0.90	1.66	1.07	9.90	1.30	4.84	0.75	9.07	1.47
45	6.92	1.19	2.02	0.87	1.66	1.11	9.95	1.30	5.52	0.55	9.51	0.93
60	7.03	1.27	2.05	0.89	1.89	1.14	10.02	1.30	5.78	0.82	9.50	1.29
90	7.09	1.21	2.11	0.92	2.21	1.17	10.02	1.30	6.70	0.40	9.67	1.04
120	7.31	1.25	2.16	0.93	2.27	1.16	10.09	1.31	6.92	0.68	9.67	1.19
150	7.33	1.26	2.22	0.97	2.33	1.15	10.16	1.33	7.31	1.07	9.89	0.85
180	7.44	1.34	2.30	0.96	2.35	1.16	10.23	1.35	7.59	1.20	9.78	1.16
210	7.57	1.44	2.36	1.01	2.37	1.15	10.25	1.36	7.69	1.28	9.89	1.05
240	7.67	1.43	2.42	0.98	2.45	1.12	10.32	1.38	8.05	1.04	9.89	1.09
270	7.69	1.41	2.47	1.02	2.46	1.11	10.35	1.39	8.18	1.02	10.00	1.11
300	7.90	1.58	2.58	0.88	2.51	1.13	10.41	1.41	8.13	1.21	10.02	1.13
330	7.94	1.57	2.67	0.84	2.57	1.14	10.49	1.47	8.49	1.23	10.09	1.06
360	7.97	1.52	2.77	0.80	2.69	1.19	10.62	1.50	8.54	1.05	10.16	1.05
1440	9.62	1.27	3.50	1.22	3.51	1.30	11.62	1.54	8.74	2.05	11.01	0.12
2880	11.22	0.87	3.57	1.14	4.22	1.70	12.06	1.57	8.64	1.60	11.43	0.33

Table S3. API release kinetic parameters of gels calculated using Baker-Lonsdale kinetic model.

API	Gel 1			Gel 2			Control		
	K_{BL} [min^{-1}*10^{-4}]		R^2	K_{BL} [min^{-1}*10^{-4}]		R^2	K_{BL} [min^{-1}*10^{-4}]		R^2
	Value	±		Value	±		Value	±	
BAC	0.03927	0.00759	0.5606	0.09287	0.02002	0.5062	0.02738	0.00644	0.4626
BG	0.01178	0.00110	0.8441	0.01491	0.00225	0.6762	0.01938	0.00630	0.3104
FUR	0.00487	0.00095	0.5566	0.00512	0.00084	0.6393	0.04824	0.01014	0.5187
GV	0.06476	0.01229	0.5694	0.07214	0.00996	0.7143	0.08232	0.01656	0.5405

Table S4. API release kinetic parameters of gels calculated using first order kinetic model.

API	Gel 1					Gel 2					Control				
	M_{eq}		K_1 [min^{-1}]		R^2	M_{eq}		K_1 [min^{-1}]		R^2	M_{eq}		K_1 [min^{-1}]		R^2
	Value	±	Value	±		Value	±	Value	±		Value	±	Value	±	
BAC	6.79969	0.04063	2.74349	0.71823	0.0775	10.3100	0.05467	2.86161	0.53764	0.1307	5.74429	0.01527	2.63997	0.16075	0.6504
BG	3.21770	0.15879	0.06087	0.01013	0.9417	4.68510	0.04996	0.33471	0.06283	0.6118	5.28732	0.04611	1.69271	0.29647	0.0979
FUR	2.14143	0.08507	0.67408	0.12055	0.4313	2.10442	0.10758	0.24112	0.05358	0.5478	7.14785	0.10792	0.46758	0.04785	0.7833
GV	6.19216	0.37194	0.16509	0.01853	0.8340	9.70976	0.09946	0.56003	0.08401	0.5654	9.95139	0.09335	1.86927	0.30536	0.2707

Table S5. API release kinetic parameters of gels calculated using second order kinetic model.

API	Gel 1					Gel 2					Control				
	M_{eq}		K_2 [%$^{-1}$min^{-1}]		R^2	M_{eq}		K_2 [%$^{-1}$min^{-1}]		R^2	M_{eq}		K_2 [%$^{-1}$min^{-1}]		R^2
	**Value**	**±**	**Value**	**±**		**Value**	**±**	**Value**	**±**		**Value**	**±**	**Value**	**±**	
BAC	6.84684	0.03703	1.21569	0.37961	0.36877	10.39095	0.04563	0.96079	0.22186	0.51486	5.76683	0.01480	2.30434	0.34826	0.71707
BG	3.63858	0.13327	0.01978	0.00310	0.97611	4.83345	0.02875	0.11976	0.01267	0.91354	5.41068	0.04529	1.20747	0.27721	0.51608
FUR	2.23371	0.07564	0.50404	0.12186	0.63613	2.25841	0.09494	0.13784	0.03456	0.74196	7.42321	0.11095	0.11813	0.01820	0.83244
GV	6.92618	0.33268	0.02611	0.00382	0.91475	9.85575	0.06591	0.10495	0.01464	0.83742	10.1111	0.06433	0.42057	0.06705	0.72163

Table S6. API release kinetic parameters of gels calculated using Korsmeyer – Peppas kinetic model.

API	Gel 1					Gel 2					Control				
	K_{KP} [min^{-1}]		n		R^2	K_{KP} [min^{-1}]		n		R^2	K_{KP} [min^{-1}]		n		R^2
	**Value**	**±**	**Value**	**±**		**Value**	**±**	**Value**	**±**		**Value**	**±**	**Value**	**±**	
BAC	6.34819	0.04595	0.01691	0.00168	0.8371	9.74710	0.03676	0.01465	0.00092	0.9279	5.58190	0.05138	0.00675	0.00229	0.3076
BG	0.46871	0.04842	0.39056	0.02170	0.9570	3.67625	0.11517	0.05066	0.00644	0.7695	5.01072	0.01756	0.01805	0.00106	0.9342
FUR	1.42371	0.03315	0.09903	0.00562	0.9403	0.97396	0.01995	0.16828	0.00445	0.9882	5.34013	0.22330	0.06799	0.01011	0.7050
GV	1.78464	0.03965	0.28170	0.00646	0.9842	7.47616	0.15653	0.05354	0.00426	0.8991	8.91039	0.04739	0.02770	0.00126	0.9614

Linear Mixed Model (LMM)

API release data were analyzed using LMM with fixed effects for sample type, time, and their interaction and a random intercept for sample ID [8]. Data were analysed using LMM for continuous *y*, normal distribution of residuals, and dependent variable scores. Degrees of freedom were estimated via the Satterthwaite method. Post-hoc comparisons tests were adjusted using Tukey's honest significant difference using JAMOVI (https://www.jamovi.org/).

Table S7. BAC release LMM fit.

Type	R^2	df	LRT X^2	p
Conditional	0.977	75	922.238	< 0.001
Marginal	0.964	74	922.264	< 0.001

R^2 (Marginal / Conditional): Proportion of variance explained by fixed effects or by the full model (including random effects).

df: Degrees of freedom used in the likelihood ratio test.

LRT X^2: Likelihood Ratio Chi-square statistic comparing full vs. reduced models.

p: p-value indicating whether including random effects significantly improves model fit.

Table S8. BAC release - Fixed Effects Omnibus Tests.

	F	df	df (res)	p
SAMPLE	2655.31	2	148	< 0.001
TIME	151.18	24	148	< 0.001
SAMPLE ✻ TIME	6.94	48	148	< 0.001

F: F-statistic testing if the effect explains significant variance.

df: Numerator degrees of freedom (effect).

df (res): Residual (denominator) degrees of freedom.

p: p-value for the F-test; significance threshold.

Table S9. BAC release - Parameter Estimates (Fixed coefficients).

Parameter Estimates (Fixed coefficients)

Names	Effect	Estimate	SE	95% Confidence Intervals Lower	95% Confidence Intervals Upper	df	t	p
(Intercept)	(Intercept)	7.430	0.1750	7.0848	7.76	2.00	42.45	<.001
SAMPLE1	BAC_GEL_1 - BAC_CONTROL	1.182	0.0649	1.0626	1.31	148.00	18.20	<.001
SAMPLE2	BAC_GEL_2 - BAC_CONTROL	4.559	0.0649	4.4283	4.68	148.00	70.21	<.001
TIME1	1 - 0	7.182	0.1874	6.8231	7.55	148.00	38.31	<.001
TIME2	2 - 0	7.277	0.1874	6.9254	7.67	148.00	38.82	<.001
TIME3	3 - 0	7.350	0.1874	6.9946	7.72	148.00	39.21	<.001
TIME4	4 - 0	7.415	0.1874	7.0507	7.79	148.00	39.56	<.001
TIME5	5 - 0	7.435	0.1874	7.0682	7.78	148.00	39.67	<.001
TIME6	10 - 0	7.492	0.1874	7.0931	7.87	148.00	39.97	<.001
TIME7	15 - 0	7.552	0.1874	7.1796	7.94	148.00	40.29	<.001
TIME8	20 - 0	7.522	0.1874	7.1818	7.90	148.00	40.13	<.001
TIME9	25 - 0	7.568	0.1874	7.1863	7.92	148.00	40.37	<.001
TIME10	30 - 0	7.588	0.1874	7.2223	7.95	148.00	40.48	<.001
TIME11	45 - 0	7.567	0.1874	7.1979	7.93	148.00	40.37	<.001
TIME12	60 - 0	7.573	0.1874	7.1910	7.94	148.00	40.40	<.001
TIME13	90 - 0	7.610	0.1874	7.2464	7.97	148.00	40.60	<.001
TIME14	120 - 0	7.641	0.1874	7.2599	8.03	148.00	40.76	<.001
TIME15	150 - 0	7.670	0.1874	7.3093	8.05	148.00	40.92	<.001
TIME16	180 - 0	7.730	0.1874	7.3630	8.09	148.00	41.24	<.001
TIME17	210 - 0	7.741	0.1874	7.3906	8.12	148.00	41.30	<.001

Table S9. BAC release - Parameter Estimates (Fixed coefficients).

Parameter Estimates (Fixed coefficients)

Names	Effect	Estimate	SE	95% Confidence Intervals		df	t	p
				Lower	Upper			
TIME18	240 - 0	7.778	0.1874	7.4264	8.17	148.00	41.50	<.001
TIME19	270 - 0	7.765	0.1874	7.3888	8.13	148.00	41.43	<.001
TIME20	300 - 0	7.806	0.1874	7.4443	8.18	148.00	41.65	<.001
TIME21	330 - 0	7.931	0.1874	7.5677	8.31	148.00	42.31	<.001
TIME22	360 - 0	7.960	0.1874	7.5940	8.31	148.00	42.46	<.001
TIME23	1440 - 0	9.101	0.1874	8.7248	9.48	148.00	48.55	<.001
TIME24	2880 - 0	9.507	0.1874	9.1204	9.87	148.00	50.72	<.001
SAMPLE1 ✻ TIME1	(BAC_GEL_1 - BAC_CONTROL) ✻ (1 - 0)	1.068	0.4591	0.1538	1.98	148.00	2.33	0.021
SAMPLE2 ✻ TIME1	(BAC_GEL_2 - BAC_CONTROL) ✻ (1 - 0)	4.444	0.4591	3.5802	5.35	148.00	9.68	<.001
SAMPLE1 ✻ TIME2	(BAC_GEL_1 - BAC_CONTROL) ✻ (2 - 0)	0.884	0.4591	0.0625	1.80	148.00	1.93	0.056
SAMPLE2 ✻ TIME2	(BAC_GEL_2 - BAC_CONTROL) ✻ (2 - 0)	4.267	0.4591	3.3809	5.13	148.00	9.29	<.001
SAMPLE1 ✻ TIME3	(BAC_GEL_1 - BAC_CONTROL) ✻ (3 - 0)	0.855	0.4591	-0.0358	1.73	148.00	1.86	0.065
SAMPLE2 ✻ TIME3	(BAC_GEL_2 - BAC_CONTROL) ✻ (3 - 0)	4.259	0.4591	3.3845	5.23	148.00	9.28	<.001
SAMPLE1 ✻ TIME4	(BAC_GEL_1 - BAC_CONTROL) ✻ (4 - 0)	0.853	0.4591	0.0570	1.72	148.00	1.86	0.065
SAMPLE2 ✻ TIME4	(BAC_GEL_2 - BAC_CONTROL) ✻ (4 - 0)	4.266	0.4591	3.3301	5.15	148.00	9.29	<.001
SAMPLE1 ✻ TIME5	(BAC_GEL_1 - BAC_CONTROL) ✻ (5 - 0)	0.952	0.4591	0.0557	1.81	148.00	2.07	0.040
SAMPLE2 ✻ TIME5	(BAC_GEL_2 - BAC_CONTROL) ✻ (5 - 0)	4.385	0.4591	3.4970	5.25	148.00	9.55	<.001
SAMPLE1 ✻ TIME6	(BAC_GEL_1 - BAC_CONTROL) ✻ (10 - 0)	0.925	0.4591	0.0670	1.78	148.00	2.02	0.046
SAMPLE2 ✻ TIME6	(BAC_GEL_2 - BAC_CONTROL) ✻ (10 - 0)	4.394	0.4591	3.4313	5.31	148.00	9.57	<.001
SAMPLE1 ✻ TIME7	(BAC_GEL_1 - BAC_CONTROL) ✻ (15 - 0)	0.810	0.4591	-0.1308	1.71	148.00	1.76	0.080

Table S9. BAC release - Parameter Estimates (Fixed coefficients).

Parameter Estimates (Fixed coefficients)

				95% Confidence Intervals				
**Names**	**Effect**	**Estimate**	**SE**	**Lower**	**Upper**	**df**	**t**	**p**
SAMPLE2 ✻ TIME7	(BAC_GEL_2 - BAC_CONTROL) ✻ (15 - 0)	4.312	0.4591	3.3167	5.18	148.00	9.39	<.001
SAMPLE1 ✻ TIME8	(BAC_GEL_1 - BAC_CONTROL) ✻ (20 - 0)	0.937	0.4591	0.0166	1.85	148.00	2.04	0.043
SAMPLE2 ✻ TIME8	(BAC_GEL_2 - BAC_CONTROL) ✻ (20 - 0)	4.461	0.4591	3.5903	5.41	148.00	9.72	<.001
SAMPLE1 ✻ TIME9	(BAC_GEL_1 - BAC_CONTROL) ✻ (25 - 0)	0.859	0.4591	0.0504	1.76	148.00	1.87	0.063
SAMPLE2 ✻ TIME9	(BAC_GEL_2 - BAC_CONTROL) ✻ (25 - 0)	4.395	0.4591	3.4703	5.28	148.00	9.57	<.001
SAMPLE1 ✻ TIME10	(BAC_GEL_1 - BAC_CONTROL) ✻ (30 - 0)	0.900	0.4591	0.0358	1.78	148.00	1.96	0.052
SAMPLE2 ✻ TIME10	(BAC_GEL_2 - BAC_CONTROL) ✻ (30 - 0)	4.444	0.4591	3.6114	5.30	148.00	9.68	<.001
SAMPLE1 ✻ TIME11	(BAC_GEL_1 - BAC_CONTROL) ✻ (45 - 0)	0.978	0.4591	0.1329	1.88	148.00	2.13	0.035
SAMPLE2 ✻ TIME11	(BAC_GEL_2 - BAC_CONTROL) ✻ (45 - 0)	4.522	0.4591	3.5894	5.44	148.00	9.85	<.001
SAMPLE1 ✻ TIME12	(BAC_GEL_1 - BAC_CONTROL) ✻ (60 - 0)	1.045	0.4591	0.1422	1.90	148.00	2.28	0.024
SAMPLE2 ✻ TIME12	(BAC_GEL_2 - BAC_CONTROL) ✻ (60 - 0)	4.596	0.4591	3.7079	5.51	148.00	10.01	<.001
SAMPLE1 ✻ TIME13	(BAC_GEL_1 - BAC_CONTROL) ✻ (90 - 0)	1.022	0.4591	0.1865	1.97	148.00	2.23	0.027
SAMPLE2 ✻ TIME13	(BAC_GEL_2 - BAC_CONTROL) ✻ (90 - 0)	4.587	0.4591	3.6770	5.47	148.00	9.99	<.001
SAMPLE1 ✻ TIME14	(BAC_GEL_1 - BAC_CONTROL) ✻ (120 - 0)	1.093	0.4591	0.2163	1.96	148.00	2.38	0.019
SAMPLE2 ✻ TIME14	(BAC_GEL_2 - BAC_CONTROL) ✻ (120 - 0)	4.666	0.4591	3.7940	5.57	148.00	10.16	<.001
SAMPLE1 ✻ TIME15	(BAC_GEL_1 - BAC_CONTROL) ✻ (150 - 0)	1.047	0.4591	0.1705	2.00	148.00	2.28	0.024
SAMPLE2 ✻ TIME15	(BAC_GEL_2 - BAC_CONTROL) ✻ (150 - 0)	4.599	0.4591	3.7010	5.53	148.00	10.02	<.001
SAMPLE1 ✻ TIME16	(BAC_GEL_1 - BAC_CONTROL) ✻ (180 - 0)	1.058	0.4591	0.1558	1.98	148.00	2.30	0.023
SAMPLE2 ✻ TIME16	(BAC_GEL_2 - BAC_CONTROL) ✻ (180 - 0)	4.647	0.4591	3.7887	5.62	148.00	10.12	<.001
SAMPLE1 ✻ TIME17	(BAC_GEL_1 - BAC_CONTROL) ✻ (210 - 0)	1.184	0.4591	0.2884	2.00	148.00	2.58	0.011

Table S9. BAC release - Parameter Estimates (Fixed coefficients).

Parameter Estimates (Fixed coefficients)

				95% Confidence Intervals				
**Names**	**Effect**	**Estimate**	**SE**	**Lower**	**Upper**	**df**	**t**	**p**
SAMPLE2 ✻ TIME17	(BAC_GEL_2 - BAC_CONTROL) ✻ (210 - 0)	4.776	0.4591	3.9251	5.66	148.00	10.40	<.001
SAMPLE1 ✻ TIME18	(BAC_GEL_1 - BAC_CONTROL) ✻ (240 - 0)	1.224	0.4591	0.2905	2.12	148.00	2.67	0.009
SAMPLE2 ✻ TIME18	(BAC_GEL_2 - BAC_CONTROL) ✻ (240 - 0)	4.815	0.4591	3.9134	5.66	148.00	10.49	<.001
SAMPLE1 ✻ TIME19	(BAC_GEL_1 - BAC_CONTROL) ✻ (270 - 0)	1.432	0.4591	0.6220	2.33	148.00	3.12	0.002
SAMPLE2 ✻ TIME19	(BAC_GEL_2 - BAC_CONTROL) ✻ (270 - 0)	5.037	0.4591	4.1670	5.94	148.00	10.97	<.001
SAMPLE1 ✻ TIME20	(BAC_GEL_1 - BAC_CONTROL) ✻ (300 - 0)	1.446	0.4591	0.4928	2.35	148.00	3.15	0.002
SAMPLE2 ✻ TIME20	(BAC_GEL_2 - BAC_CONTROL) ✻ (300 - 0)	5.048	0.4591	4.1916	5.95	148.00	10.99	<.001
SAMPLE1 ✻ TIME21	(BAC_GEL_1 - BAC_CONTROL) ✻ (330 - 0)	1.272	0.4591	0.3800	2.15	148.00	2.77	0.006
SAMPLE2 ✻ TIME21	(BAC_GEL_2 - BAC_CONTROL) ✻ (330 - 0)	4.890	0.4591	3.9953	5.84	148.00	10.65	<.001
SAMPLE1 ✻ TIME22	(BAC_GEL_1 - BAC_CONTROL) ✻ (360 - 0)	1.450	0.4591	0.5895	2.31	148.00	3.16	0.002
SAMPLE2 ✻ TIME22	(BAC_GEL_2 - BAC_CONTROL) ✻ (360 - 0)	5.068	0.4591	4.1672	5.95	148.00	11.04	<.001
SAMPLE1 ✻ TIME23	(BAC_GEL_1 - BAC_CONTROL) ✻ (1440 - 0)	2.752	0.4591	1.9036	3.63	148.00	5.99	<.001
SAMPLE2 ✻ TIME23	(BAC_GEL_2 - BAC_CONTROL) ✻ (1440 - 0)	6.349	0.4591	5.4923	7.24	148.00	13.83	<.001
SAMPLE1 ✻ TIME24	(BAC_GEL_1 - BAC_CONTROL) ✻ (2880 - 0)	3.495	0.4591	2.6383	4.39	148.00	7.61	<.001
SAMPLE2 ✻ TIME24	(BAC_GEL_2 - BAC_CONTROL) ✻ (2880 - 0)	6.747	0.4591	5.8575	7.65	148.00	14.70	<.001

Effect: Predictor term.
Estimate (B): Estimated effect size; expected change in the dependent variable associated with a one-unit increase in the predictor.
SE (Standard Error): Precision of the estimate; lower values indicate greater reliability.
df: Degrees of freedom associated with the t-test for the effect.
t: Test statistic indicating how far the estimate is from zero, in standard error units.
p: p-value assessing the statistical significance of the effect.

Table S10. BAC release – Random components.

Groups	Name	Variance	SD	ICC
ID	(Intercept)	0.0898	0.300	0.362
Residual		0.1581	0.398	

Variance: Estimated variance for the random effect (e.g., subject-level variability).
SD (Standard Deviation): Square root of the variance, indicating the spread of random intercepts.
ICC (Intraclass Correlation Coefficient): Proportion of total variance explained by between-subject differences. Values closer to 1 indicate high within-subject consistency across repeated measures.

Table S11. BAC release – Post hoc comparison: SAMPLE.

Comparison							
SAMPLE	*vs*	SAMPLE	Difference	SE	t	df	p_{tukey}
BAC_CONTROL	-	BAC_GEL_1	-1.18	0.0649	-18.2	148	<0.001
BAC_CONTROL	-	BAC_GEL_2	-4.56	0.0649	-70.2	148	<0.001
BAC_GEL_1	-	BAC_GEL_2	-3.38	0.0649	-52.0	148	<0.001

Difference: Estimated mean difference between two levels of the factor.
SE (Standard Error): Standard error of the estimated difference.
t: Test statistic for the contrast.
df: Degrees of freedom for the comparison.
p Tukey: p-value adjusted using Tukey's method.

Table S12. BG release LMM fit.

Type	R^2	df	LRT X^2	p
Conditional	0.959	75	794.413	< 0.001
Marginal	0.930	74	794.316	< 0.001

R^2 (Marginal / Conditional): Proportion of variance explained by fixed effects or by the full model (including random effects).
df: Degrees of freedom used in the likelihood ratio test.
LRT X^2: Likelihood Ratio Chi-square statistic comparing full vs. reduced models.
p: p-value indicating whether including random effects significantly improves model fit.

Table S13. BG release - Fixed Effects Omnibus Tests.

	F	df	df (res)	p
SAMPLE	1155.1	2	148	< 0.001
TIME	96.1	24	148	< 0.001
SAMPLE ✻ TIME	11.0	48	148	< 0.001

F: F-statistic testing if the effect explains significant variance.
df: Numerator degrees of freedom (effect).
df (res): Residual (denominator) degrees of freedom.
p: p-value for the F-test; significance threshold.

Table S14. BG release - Parameter Estimates (Fixed coefficients).

Parameter Estimates (Fixed coefficients)

Names	Effect	Estimate	SE	95% Confidence Intervals		df	t	p
				Lower	Upper			
(Intercept)	(Intercept)	3.952	0.1649	3.64	4.27228	2.00	23.97	0.002
SAMPLE1	BG_GEL_1 - BG_CONTROL	-2.597	0.0546	-2.71	-2.49028	148.00	-47.58	<.001
SAMPLE2	BG_GEL_2 - BG_CONTROL	-0.976	0.0546	-1.09	-0.86824	148.00	-17.89	<.001
TIME1	1 - 0	2.443	0.1575	2.14	2.74262	148.00	15.51	<.001
TIME2	2 - 0	2.858	0.1575	2.57	3.16434	148.00	18.14	<.001
TIME3	3 - 0	3.024	0.1575	2.73	3.32862	148.00	19.20	<.001
TIME4	4 - 0	3.204	0.1575	2.89	3.49033	148.00	20.34	<.001
TIME5	5 - 0	3.344	0.1575	3.03	3.63388	148.00	21.23	<.001
TIME6	10 - 0	3.630	0.1575	3.34	3.94086	148.00	23.04	<.001
TIME7	15 - 0	3.778	0.1575	3.48	4.09193	148.00	23.98	<.001
TIME8	20 - 0	3.863	0.1575	3.56	4.14395	148.00	24.52	<.001
TIME9	25 - 0	3.929	0.1575	3.63	4.22859	148.00	24.94	<.001
TIME10	30 - 0	3.998	0.1575	3.70	4.29904	148.00	25.38	<.001
TIME11	45 - 0	4.097	0.1575	3.80	4.40470	148.00	26.00	<.001
TIME12	60 - 0	4.136	0.1575	3.85	4.44836	148.00	26.25	<.001
TIME13	90 - 0	4.272	0.1575	3.97	4.55826	148.00	27.12	<.001
TIME14	120 - 0	4.350	0.1575	4.06	4.65802	148.00	27.61	<.001
TIME15	150 - 0	4.509	0.1575	4.20	4.81746	148.00	28.62	<.001
TIME16	180 - 0	4.563	0.1575	4.26	4.85212	148.00	28.97	<.001
TIME17	210 - 0	4.669	0.1575	4.39	4.98874	148.00	29.64	<.001

Table S14. BG release - Parameter Estimates (Fixed coefficients).

Parameter Estimates (Fixed coefficients)

Names	Effect	Estimate	SE	95% Confidence Intervals Lower	Upper	df	t	p
TIME18	240 - 0	4.758	0.1575	4.46	5.07060	148.00	30.20	<.001
TIME19	270 - 0	4.784	0.1575	4.48	5.09733	148.00	30.37	<.001
TIME20	300 - 0	4.812	0.1575	4.52	5.09961	148.00	30.54	<.001
TIME21	330 - 0	4.841	0.1575	4.52	5.13906	148.00	30.73	<.001
TIME22	360 - 0	4.877	0.1575	4.59	5.17777	148.00	30.95	<.001
TIME23	1440 - 0	4.968	0.1575	4.67	5.27827	148.00	31.54	<.001
TIME24	2880 - 0	5.098	0.1575	4.79	5.40638	148.00	32.36	<.001
SAMPLE1 ✻ TIME1	(BG_GEL_1 - BG_CONTROL) ✻ (1 - 0)	-4.765	0.3859	-5.53	-4.02309	148.00	-12.35	<.001
SAMPLE2 ✻ TIME1	(BG_GEL_2 - BG_CONTROL) ✻ (1 - 0)	-3.055	0.3859	-3.81	-2.30139	148.00	-7.92	<.001
SAMPLE1 ✻ TIME2	(BG_GEL_1 - BG_CONTROL) ✻ (2 - 0)	-4.486	0.3859	-5.15	-3.64554	148.00	-11.62	<.001
SAMPLE2 ✻ TIME2	(BG_GEL_2 - BG_CONTROL) ✻ (2 - 0)	-2.322	0.3859	-3.08	-1.56651	148.00	-6.02	<.001
SAMPLE1 ✻ TIME3	(BG_GEL_1 - BG_CONTROL) ✻ (3 - 0)	-4.319	0.3859	-5.08	-3.54963	148.00	-11.19	<.001
SAMPLE2 ✻ TIME3	(BG_GEL_2 - BG_CONTROL) ✻ (3 - 0)	-1.989	0.3859	-2.72	-1.29458	148.00	-5.15	<.001
SAMPLE1 ✻ TIME4	(BG_GEL_1 - BG_CONTROL) ✻ (4 - 0)	-4.136	0.3859	-4.92	-3.36322	148.00	-10.72	<.001
SAMPLE2 ✻ TIME4	(BG_GEL_2 - BG_CONTROL) ✻ (4 - 0)	-1.671	0.3859	-2.42	-0.92278	148.00	-4.33	<.001
SAMPLE1 ✻ TIME5	(BG_GEL_1 - BG_CONTROL) ✻ (5 - 0)	-4.099	0.3859	-4.85	-3.37596	148.00	-10.62	<.001
SAMPLE2 ✻ TIME5	(BG_GEL_2 - BG_CONTROL) ✻ (5 - 0)	-1.319	0.3859	-2.00	-0.56489	148.00	-3.42	<.001
SAMPLE1 ✻ TIME6	(BG_GEL_1 - BG_CONTROL) ✻ (10 - 0)	-3.635	0.3859	-4.38	-2.82707	148.00	-9.42	<.001
SAMPLE2 ✻ TIME6	(BG_GEL_2 - BG_CONTROL) ✻ (10 - 0)	-0.975	0.3859	-1.70	-0.20094	148.00	-2.53	0.013
SAMPLE1 ✻ TIME7	(BG_GEL_1 - BG_CONTROL) ✻ (15 - 0)	-3.356	0.3859	-4.07	-2.59281	148.00	-8.70	<.001

Table S14. BG release - Parameter Estimates (Fixed coefficients).

Parameter Estimates (Fixed coefficients)

				95% Confidence Intervals				
Names	Effect	Estimate	SE	Lower	Upper	df	t	p
SAMPLE2 ✻ TIME7	(BG_GEL_2 - BG_CONTROL) ✻ (15 - 0)	-0.810	0.3859	-1.56	-0.03446	148.00	-2.10	0.038
SAMPLE1 ✻ TIME8	(BG_GEL_1 - BG_CONTROL) ✻ (20 - 0)	-3.179	0.3859	-3.92	-2.47441	148.00	-8.24	<.001
SAMPLE2 ✻ TIME8	(BG_GEL_2 - BG_CONTROL) ✻ (20 - 0)	-0.772	0.3859	-1.49	-0.05108	148.00	-2.00	0.047
SAMPLE1 ✻ TIME9	(BG_GEL_1 - BG_CONTROL) ✻ (25 - 0)	-3.007	0.3859	-3.67	-2.26698	148.00	-7.79	<.001
SAMPLE2 ✻ TIME9	(BG_GEL_2 - BG_CONTROL) ✻ (25 - 0)	-0.763	0.3859	-1.43	-0.09134	148.00	-1.98	0.050
SAMPLE1 ✻ TIME10	(BG_GEL_1 - BG_CONTROL) ✻ (30 - 0)	-3.075	0.3859	-3.80	-2.33062	148.00	-7.97	<.001
SAMPLE2 ✻ TIME10	(BG_GEL_2 - BG_CONTROL) ✻ (30 - 0)	-0.869	0.3859	-1.62	-0.17001	148.00	-2.25	0.026
SAMPLE1 ✻ TIME11	(BG_GEL_1 - BG_CONTROL) ✻ (45 - 0)	-2.903	0.3859	-3.62	-2.15776	148.00	-7.52	<.001
SAMPLE2 ✻ TIME11	(BG_GEL_2 - BG_CONTROL) ✻ (45 - 0)	-0.765	0.3859	-1.51	-0.05338	148.00	-1.98	0.049
SAMPLE1 ✻ TIME12	(BG_GEL_1 - BG_CONTROL) ✻ (60 - 0)	-2.863	0.3859	-3.64	-2.07190	148.00	-7.42	<.001
SAMPLE2 ✻ TIME12	(BG_GEL_2 - BG_CONTROL) ✻ (60 - 0)	-0.746	0.3859	-1.51	0.01107	148.00	-1.93	0.055
SAMPLE1 ✻ TIME13	(BG_GEL_1 - BG_CONTROL) ✻ (90 - 0)	-2.490	0.3859	-3.25	-1.71892	148.00	-6.45	<.001
SAMPLE2 ✻ TIME13	(BG_GEL_2 - BG_CONTROL) ✻ (90 - 0)	-0.729	0.3859	-1.49	-0.00243	148.00	-1.89	0.061
SAMPLE1 ✻ TIME14	(BG_GEL_1 - BG_CONTROL) ✻ (120 - 0)	-2.282	0.3859	-3.08	-1.51472	148.00	-5.91	<.001
SAMPLE2 ✻ TIME14	(BG_GEL_2 - BG_CONTROL) ✻ (120 - 0)	-0.723	0.3859	-1.44	-0.01358	148.00	-1.87	0.063
SAMPLE1 ✻ TIME15	(BG_GEL_1 - BG_CONTROL) ✻ (150 - 0)	-1.973	0.3859	-2.72	-1.23509	148.00	-5.11	<.001
SAMPLE2 ✻ TIME15	(BG_GEL_2 - BG_CONTROL) ✻ (150 - 0)	-0.782	0.3859	-1.52	-0.02183	148.00	-2.03	0.044
SAMPLE1 ✻ TIME16	(BG_GEL_1 - BG_CONTROL) ✻ (180 - 0)	-1.887	0.3859	-2.60	-1.13769	148.00	-4.89	<.001
SAMPLE2 ✻ TIME16	(BG_GEL_2 - BG_CONTROL) ✻ (180 - 0)	-0.789	0.3859	-1.54	-0.08565	148.00	-2.04	0.043
SAMPLE1 ✻ TIME17	(BG_GEL_1 - BG_CONTROL) ✻ (210 - 0)	-1.813	0.3859	-2.57	-1.06789	148.00	-4.70	<.001

Table S14. BG release - Parameter Estimates (Fixed coefficients).

Parameter Estimates (Fixed coefficients)

				95% Confidence Intervals				
Names	Effect	Estimate	SE	Lower	Upper	df	t	p
SAMPLE2 ✻ TIME17	(BG_GEL_2 - BG_CONTROL) ✻ (210 - 0)	-0.857	0.3859	-1.60	-0.13428	148.00	-2.22	0.028
SAMPLE1 ✻ TIME18	(BG_GEL_1 - BG_CONTROL) ✻ (240 - 0)	-1.713	0.3859	-2.39	-0.96503	148.00	-4.44	<.001
SAMPLE2 ✻ TIME18	(BG_GEL_2 - BG_CONTROL) ✻ (240 - 0)	-0.729	0.3859	-1.46	0.04772	148.00	-1.89	0.061
SAMPLE1 ✻ TIME19	(BG_GEL_1 - BG_CONTROL) ✻ (270 - 0)	-1.660	0.3859	-2.45	-0.95158	148.00	-4.30	<.001
SAMPLE2 ✻ TIME19	(BG_GEL_2 - BG_CONTROL) ✻ (270 - 0)	-0.704	0.3859	-1.46	-0.04539	148.00	-1.82	0.070
SAMPLE1 ✻ TIME20	(BG_GEL_1 - BG_CONTROL) ✻ (300 - 0)	-1.612	0.3859	-2.34	-0.84260	148.00	-4.18	<.001
SAMPLE2 ✻ TIME20	(BG_GEL_2 - BG_CONTROL) ✻ (300 - 0)	-0.668	0.3859	-1.40	0.08851	148.00	-1.73	0.086
SAMPLE1 ✻ TIME21	(BG_GEL_1 - BG_CONTROL) ✻ (330 - 0)	-1.556	0.3859	-2.30	-0.81657	148.00	-4.03	<.001
SAMPLE2 ✻ TIME21	(BG_GEL_2 - BG_CONTROL) ✻ (330 - 0)	-0.683	0.3859	-1.40	0.01416	148.00	-1.77	0.079
SAMPLE1 ✻ TIME22	(BG_GEL_1 - BG_CONTROL) ✻ (360 - 0)	-1.503	0.3859	-2.23	-0.76710	148.00	-3.89	<.001
SAMPLE2 ✻ TIME22	(BG_GEL_2 - BG_CONTROL) ✻ (360 - 0)	-0.647	0.3859	-1.37	0.06785	148.00	-1.68	0.096
SAMPLE1 ✻ TIME23	(BG_GEL_1 - BG_CONTROL) ✻ (1440 - 0)	-1.355	0.3859	-2.09	-0.55938	148.00	-3.51	<.001
SAMPLE2 ✻ TIME23	(BG_GEL_2 - BG_CONTROL) ✻ (1440 - 0)	-0.628	0.3859	-1.38	0.05907	148.00	-1.63	0.106
SAMPLE1 ✻ TIME24	(BG_GEL_1 - BG_CONTROL) ✻ (2880 - 0)	-1.245	0.3859	-2.00	-0.53745	148.00	-3.23	0.002
SAMPLE2 ✻ TIME24	(BG_GEL_2 - BG_CONTROL) ✻ (2880 - 0)	-0.411	0.3859	-1.20	0.36995	148.00	-1.07	0.288

Effect: Predictor term.
Estimate (B): Estimated effect size; expected change in the dependent variable associated with a one-unit increase in the predictor.
SE (Standard Error): Precision of the estimate; lower values indicate greater reliability.
df: Degrees of freedom associated with the t-test for the effect.
t: Test statistic indicating how far the estimate is from zero, in standard error units.
p: p-value assessing the statistical significance of the effect.

Table S15. BG release – Random components.

Groups	Name	Variance	SD	ICC
ID	(Intercept)	0.0801	0.283	0.418
Residual		0.1117	0.334	

Variance: Estimated variance for the random effect (e.g., subject-level variability).
SD (Standard Deviation): Square root of the variance, indicating the spread of random intercepts.
ICC (Intraclass Correlation Coefficient): Proportion of total variance explained by between-subject differences. Values closer to 1 indicate high within-subject consistency across repeated measures.

Table S16. BG release – Post hoc comparison: SAMPLE.

Comparison							
SAMPLE	*vs*	SAMPLE	Difference	SE	t	df	p_{tukey}
BG_CONTROL	-	BG_GEL_1	2.597	0.0546	47.6	148	<0.001
BG_CONTROL	-	BG_GEL_2	0.976	0.0546	17.9	148	<0.001
BG_GEL_1	-	BG_GEL_2	-1.620	0.0546	-29.7	148	<0.001

Difference: Estimated mean difference between two levels of the factor.
SE (Standard Error): Standard error of the estimated difference.
t: Test statistic for the contrast.
df: Degrees of freedom for the comparison.
p Tukey: p-value adjusted using Tukey's method.

Table S17. FUR release LMM fit.

Type	R^2	df	LRT X^2	p
Conditional	0.864	75	520.018	< 0.001
Marginal	0.847	74	520.232	< 0.001

R^2 (Marginal / Conditional): Proportion of variance explained by fixed effects or by the full model (including random effects).
df: Degrees of freedom used in the likelihood ratio test.
LRT X^2: Likelihood Ratio Chi-square statistic comparing full vs. reduced models.
p: p-value indicating whether including random effects significantly improves model fit.

Table S18. FUR release - Fixed Effects Omnibus Tests.

	F	df	df (res)	p
SAMPLE	502.71	2	148	< 0.001
TIME	11.97	24	148	< 0.001
SAMPLE ✻ TIME	2.07	48	148	< 0.001

F: F-statistic testing if the effect explains significant variance.
df: Numerator degrees of freedom (effect).
df (res): Residual (denominator) degrees of freedom.
p: p-value for the F-test; significance threshold.

Table S19. FUR release - Parameter Estimates (Fixed coefficients).

Parameter Estimates (Fixed coefficients)

Names	Effect	Estimate	SE	95% Confidence Intervals Lower	95% Confidence Intervals Upper	df	t	p
(Intercept)	(Intercept)	3.605	0.226	3.133	4.0183	2.00	15.925	0.004
SAMPLE1	FUR_GEL_1 - FUR_CONTROL	-4.627	0.171	-4.959	-4.3028	148.00	-27.039	<.001
SAMPLE2	FUR_GEL_2 - FUR_CONTROL	-4.768	0.171	-5.083	-4.4122	148.00	-27.863	<.001
TIME1	1 - 0	1.614	0.494	0.640	2.5415	148.00	3.268	0.001
TIME2	2 - 0	2.126	0.494	1.162	3.0484	148.00	4.303	<.001
TIME3	3 - 0	2.864	0.494	1.890	3.8233	148.00	5.798	<.001
TIME4	4 - 0	3.063	0.494	2.137	4.0516	148.00	6.201	<.001
TIME5	5 - 0	3.128	0.494	2.225	4.0939	148.00	6.333	<.001
TIME6	10 - 0	3.223	0.494	2.245	4.2750	148.00	6.523	<.001
TIME7	15 - 0	3.274	0.494	2.339	4.2838	148.00	6.627	<.001
TIME8	20 - 0	3.397	0.494	2.468	4.3707	148.00	6.876	<.001
TIME9	25 - 0	3.448	0.494	2.553	4.4083	148.00	6.979	<.001
TIME10	30 - 0	3.519	0.494	2.585	4.4740	148.00	7.123	<.001
TIME11	45 - 0	3.534	0.494	2.550	4.4848	148.00	7.154	<.001
TIME12	60 - 0	3.659	0.494	2.738	4.6193	148.00	7.407	<.001
TIME13	90 - 0	3.803	0.494	2.773	4.7647	148.00	7.697	<.001
TIME14	120 - 0	3.911	0.494	2.961	4.8553	148.00	7.917	<.001
TIME15	150 - 0	3.958	0.494	3.020	5.0017	148.00	8.012	<.001
TIME16	180 - 0	4.032	0.494	3.046	4.9485	148.00	8.161	<.001
TIME17	210 - 0	4.103	0.494	3.186	5.0762	148.00	8.306	<.001

Table S19. FUR release - Parameter Estimates (Fixed coefficients).

Parameter Estimates (Fixed coefficients)

				95% Confidence Intervals				
Names	Effect	Estimate	SE	Lower	Upper	df	t	p
TIME18	240 - 0	4.179	0.494	3.248	5.1674	148.00	8.459	<.001
TIME19	270 - 0	4.208	0.494	3.260	5.1906	148.00	8.518	<.001
TIME20	300 - 0	4.331	0.494	3.367	5.3057	148.00	8.767	<.001
TIME21	330 - 0	4.396	0.494	3.382	5.4448	148.00	8.899	<.001
TIME22	360 - 0	4.473	0.494	3.545	5.4309	148.00	9.055	<.001
TIME23	1440 - 0	5.545	0.494	4.636	6.5502	148.00	11.224	<.001
TIME24	2880 - 0	6.336	0.494	5.397	7.2550	148.00	12.826	<.001
SAMPLE1 ✻ TIME1	(FUR_GEL_1 - FUR_CONTROL) ✻ (1 - 0)	-0.704	1.210	-2.942	1.4962	148.00	-0.582	0.562
SAMPLE2 ✻ TIME1	(FUR_GEL_2 - FUR_CONTROL) ✻ (1 - 0)	-1.217	1.210	-3.427	1.0676	148.00	-1.005	0.316
SAMPLE1 ✻ TIME2	(FUR_GEL_1 - FUR_CONTROL) ✻ (2 - 0)	-2.316	1.210	-4.696	0.0434	148.00	-1.914	0.058
SAMPLE2 ✻ TIME2	(FUR_GEL_2 - FUR_CONTROL) ✻ (2 - 0)	-2.670	1.210	-5.065	-0.3193	148.00	-2.207	0.029
SAMPLE1 ✻ TIME3	(FUR_GEL_1 - FUR_CONTROL) ✻ (3 - 0)	-3.926	1.210	-6.309	-1.6204	148.00	-3.244	0.001
SAMPLE2 ✻ TIME3	(FUR_GEL_2 - FUR_CONTROL) ✻ (3 - 0)	-4.435	1.210	-6.838	-2.1095	148.00	-3.665	<.001
SAMPLE1 ✻ TIME4	(FUR_GEL_1 - FUR_CONTROL) ✻ (4 - 0)	-4.444	1.210	-6.800	-2.0722	148.00	-3.673	<.001
SAMPLE2 ✻ TIME4	(FUR_GEL_2 - FUR_CONTROL) ✻ (4 - 0)	-4.986	1.210	-7.424	-2.5447	148.00	-4.121	<.001
SAMPLE1 ✻ TIME5	(FUR_GEL_1 - FUR_CONTROL) ✻ (5 - 0)	-4.635	1.210	-7.008	-2.2312	148.00	-3.831	<.001
SAMPLE2 ✻ TIME5	(FUR_GEL_2 - FUR_CONTROL) ✻ (5 - 0)	-5.173	1.210	-7.612	-2.7972	148.00	-4.275	<.001
SAMPLE1 ✻ TIME6	(FUR_GEL_1 - FUR_CONTROL) ✻ (10 - 0)	-4.692	1.210	-7.089	-2.3600	148.00	-3.878	<.001
SAMPLE2 ✻ TIME6	(FUR_GEL_2 - FUR_CONTROL) ✻ (10 - 0)	-5.042	1.210	-7.439	-2.5425	148.00	-4.167	<.001
SAMPLE1 ✻ TIME7	(FUR_GEL_1 - FUR_CONTROL) ✻ (15 - 0)	-4.723	1.210	-7.064	-2.1734	148.00	-3.903	<.001

Table S19. FUR release - Parameter Estimates (Fixed coefficients).

Parameter Estimates (Fixed coefficients)

				95% Confidence Intervals				
Names	Effect	Estimate	SE	Lower	Upper	df	t	p
SAMPLE2 ✻ TIME7	(FUR_GEL_2 - FUR_CONTROL) ✻ (15 - 0)	-4.987	1.210	-7.234	-2.5597	148.00	-4.121	<.001
SAMPLE1 ✻ TIME8	(FUR_GEL_1 - FUR_CONTROL) ✻ (20 - 0)	-4.844	1.210	-7.091	-2.5834	148.00	-4.003	<.001
SAMPLE2 ✻ TIME8	(FUR_GEL_2 - FUR_CONTROL) ✻ (20 - 0)	-5.086	1.210	-7.537	-2.5331	148.00	-4.203	<.001
SAMPLE1 ✻ TIME9	(FUR_GEL_1 - FUR_CONTROL) ✻ (25 - 0)	-4.935	1.210	-7.314	-2.6091	148.00	-4.079	<.001
SAMPLE2 ✻ TIME9	(FUR_GEL_2 - FUR_CONTROL) ✻ (25 - 0)	-5.140	1.210	-7.493	-2.5937	148.00	-4.248	<.001
SAMPLE1 ✻ TIME10	(FUR_GEL_1 - FUR_CONTROL) ✻ (30 - 0)	-4.945	1.210	-7.207	-2.6092	148.00	-4.087	<.001
SAMPLE2 ✻ TIME10	(FUR_GEL_2 - FUR_CONTROL) ✻ (30 - 0)	-5.259	1.210	-7.727	-2.8165	148.00	-4.346	<.001
SAMPLE1 ✻ TIME11	(FUR_GEL_1 - FUR_CONTROL) ✻ (45 - 0)	-4.908	1.210	-7.265	-2.6500	148.00	-4.056	<.001
SAMPLE2 ✻ TIME11	(FUR_GEL_2 - FUR_CONTROL) ✻ (45 - 0)	-5.263	1.210	-7.560	-2.8550	148.00	-4.349	<.001
SAMPLE1 ✻ TIME12	(FUR_GEL_1 - FUR_CONTROL) ✻ (60 - 0)	-4.977	1.210	-7.504	-2.5922	148.00	-4.113	<.001
SAMPLE2 ✻ TIME12	(FUR_GEL_2 - FUR_CONTROL) ✻ (60 - 0)	-5.138	1.210	-7.664	-2.8309	148.00	-4.246	<.001
SAMPLE1 ✻ TIME13	(FUR_GEL_1 - FUR_CONTROL) ✻ (90 - 0)	-4.983	1.210	-7.376	-2.7433	148.00	-4.118	<.001
SAMPLE2 ✻ TIME13	(FUR_GEL_2 - FUR_CONTROL) ✻ (90 - 0)	-4.882	1.210	-7.163	-2.5517	148.00	-4.034	<.001
SAMPLE1 ✻ TIME14	(FUR_GEL_1 - FUR_CONTROL) ✻ (120 - 0)	-5.151	1.210	-7.500	-2.6732	148.00	-4.257	<.001
SAMPLE2 ✻ TIME14	(FUR_GEL_2 - FUR_CONTROL) ✻ (120 - 0)	-5.041	1.210	-7.315	-2.6992	148.00	-4.166	<.001
SAMPLE1 ✻ TIME15	(FUR_GEL_1 - FUR_CONTROL) ✻ (150 - 0)	-5.109	1.210	-7.399	-2.6048	148.00	-4.222	<.001
SAMPLE2 ✻ TIME15	(FUR_GEL_2 - FUR_CONTROL) ✻ (150 - 0)	-5.004	1.210	-7.367	-2.5096	148.00	-4.135	<.001
SAMPLE1 ✻ TIME16	(FUR_GEL_1 - FUR_CONTROL) ✻ (180 - 0)	-5.145	1.210	-7.365	-2.7485	148.00	-4.252	<.001
SAMPLE2 ✻ TIME16	(FUR_GEL_2 - FUR_CONTROL) ✻ (180 - 0)	-5.093	1.210	-7.567	-2.6996	148.00	-4.208	<.001
SAMPLE1 ✻ TIME17	(FUR_GEL_1 - FUR_CONTROL) ✻ (210 - 0)	-5.214	1.210	-7.466	-2.6693	148.00	-4.308	<.001

Table S19. FUR release - Parameter Estimates (Fixed coefficients).

Parameter Estimates (Fixed coefficients)

Names	Effect	Estimate	SE	95% Confidence Intervals		df	t	p
				Lower	Upper			
SAMPLE2 ✻ TIME17	(FUR_GEL_2 - FUR_CONTROL) ✻ (210 - 0)	-5.201	1.210	-7.584	-2.8748	148.00	-4.298	<.001
SAMPLE1 ✻ TIME18	(FUR_GEL_1 - FUR_CONTROL) ✻ (240 - 0)	-5.252	1.210	-7.673	-2.9681	148.00	-4.340	<.001
SAMPLE2 ✻ TIME18	(FUR_GEL_2 - FUR_CONTROL) ✻ (240 - 0)	-5.226	1.210	-7.590	-2.8915	148.00	-4.319	<.001
SAMPLE1 ✻ TIME19	(FUR_GEL_1 - FUR_CONTROL) ✻ (270 - 0)	-5.222	1.210	-7.434	-2.7982	148.00	-4.316	<.001
SAMPLE2 ✻ TIME19	(FUR_GEL_2 - FUR_CONTROL) ✻ (270 - 0)	-5.229	1.210	-7.617	-2.6604	148.00	-4.321	<.001
SAMPLE1 ✻ TIME20	(FUR_GEL_1 - FUR_CONTROL) ✻ (300 - 0)	-5.317	1.210	-7.652	-2.8869	148.00	-4.394	<.001
SAMPLE2 ✻ TIME20	(FUR_GEL_2 - FUR_CONTROL) ✻ (300 - 0)	-5.387	1.210	-7.829	-2.8556	148.00	-4.451	<.001
SAMPLE1 ✻ TIME21	(FUR_GEL_1 - FUR_CONTROL) ✻ (330 - 0)	-5.268	1.210	-7.653	-2.7524	148.00	-4.353	<.001
SAMPLE2 ✻ TIME21	(FUR_GEL_2 - FUR_CONTROL) ✻ (330 - 0)	-5.367	1.210	-7.772	-2.8525	148.00	-4.435	<.001
SAMPLE1 ✻ TIME22	(FUR_GEL_1 - FUR_CONTROL) ✻ (360 - 0)	-5.197	1.210	-7.463	-2.9667	148.00	-4.295	<.001
SAMPLE2 ✻ TIME22	(FUR_GEL_2 - FUR_CONTROL) ✻ (360 - 0)	-5.280	1.210	-7.669	-2.8892	148.00	-4.363	<.001
SAMPLE1 ✻ TIME23	(FUR_GEL_1 - FUR_CONTROL) ✻ (1440 - 0)	-6.119	1.210	-8.442	-3.7529	148.00	-5.056	<.001
SAMPLE2 ✻ TIME23	(FUR_GEL_2 - FUR_CONTROL) ✻ (1440 - 0)	-6.105	1.210	-8.533	-3.7808	148.00	-5.045	<.001
SAMPLE1 ✻ TIME24	(FUR_GEL_1 - FUR_CONTROL) ✻ (2880 - 0)	-7.652	1.210	-9.967	-5.2968	148.00	-6.324	<.001
SAMPLE2 ✻ TIME24	(FUR_GEL_2 - FUR_CONTROL) ✻ (2880 - 0)	-6.997	1.210	-9.341	-4.4292	148.00	-5.782	<.001

Effect: Predictor term.
Estimate (B): Estimated effect size; expected change in the dependent variable associated with a one-unit increase in the predictor.
SE (Standard Error): Precision of the estimate; lower values indicate greater reliability.
df: Degrees of freedom associated with the t-test for the effect.
t: Test statistic indicating how far the estimate is from zero, in standard error units.
p: p-value assessing the statistical significance of the effect.

Table S20. FUR release – Random components.

Groups	Name	Variance	SD	ICC
ID	(Intercept)	0.139	0.373	0.112
Residual		1.098	1.048	

Variance: Estimated variance for the random effect (e.g., subject-level variability).
SD (Standard Deviation): Square root of the variance, indicating the spread of random intercepts.
ICC (Intraclass Correlation Coefficient): Proportion of total variance explained by between-subject differences. Values closer to 1 indicate high within-subject consistency across repeated measures.

Table S21. FUR release – Post hoc comparison: SAMPLE.

Comparison							
SAMPLE	*vs*	SAMPLE	Difference	SE	t	df	p_{tukey}
FUR_CONTROL	-	FUR_GEL_1	4.627	0.171	27.039	148	<0.001
FUR_CONTROL	-	FUR_GEL_2	4.768	0.171	27.863	148	<0.001
FUR_GEL_1	-	FUR_GEL_2	0.141	0.171	0.824	148	<0.001

Difference: Estimated mean difference between two levels of the factor.
SE (Standard Error): Standard error of the estimated difference.
t: Test statistic for the contrast.
df: Degrees of freedom for the comparison.
p Tukey: p-value adjusted using Tukey's method.

Table S22. GV release LMM fit.

Type	R^2	df	LRT X^2	p
Conditional	0.860	75	508.596	< 0.001
Marginal	0.817	74	507.267	< 0.001

R^2 (Marginal / Conditional): Proportion of variance explained by fixed effects or by the full model (including random effects).
df: Degrees of freedom used in the likelihood ratio test.
LRT X^2: Likelihood Ratio Chi-square statistic comparing full vs. reduced models.
p: p-value indicating whether including random effects significantly improves model fit.

Table S23. GV release - Fixed Effects Omnibus Tests.

	F	df	df (res)	p
SAMPLE	241.14	2	148	< 0.001
TIME	30.02	24	148	< 0.001
SAMPLE ✻ TIME	2.13	48	148	< 0.001

F: F-statistic testing if the effect explains significant variance.
df: Numerator degrees of freedom (effect).
df (res): Residual (denominator) degrees of freedom.
p: p-value for the F-test; significance threshold.

Table S24. GV release - Parameter Estimates (Fixed coefficients).

Parameter Estimates (Fixed coefficients)

				95% Confidence Intervals				
Names	Effect	Estimate	SE	Lower	Upper	df	t	p
(Intercept)	(Intercept)	8.030	0.385	7.31	8.75779	2.00	20.871	0.002
SAMPLE1	GV_GEL_1 - GV_CONTROL	-4.019	0.194	-4.37	-3.59848	148.00	-20.718	<.001
SAMPLE2	GV_GEL_2 - GV_CONTROL	-0.786	0.194	-1.17	-0.41623	148.00	-4.050	<.001
TIME1	1 - 0	5.518	0.560	4.35	6.60753	148.00	9.853	<.001
TIME2	2 - 0	5.903	0.560	4.84	7.06206	148.00	10.542	<.001
TIME3	3 - 0	6.378	0.560	5.25	7.44041	148.00	11.390	<.001
TIME4	4 - 0	6.672	0.560	5.51	7.79039	148.00	11.914	<.001
TIME5	5 - 0	6.948	0.560	5.81	8.02464	148.00	12.408	<.001
TIME6	10 - 0	7.279	0.560	6.24	8.41543	148.00	12.999	<.001
TIME7	15 - 0	7.535	0.560	6.45	8.67670	148.00	13.456	<.001
TIME8	20 - 0	7.635	0.560	6.53	8.78719	148.00	13.634	<.001
TIME9	25 - 0	7.829	0.560	6.72	8.89933	148.00	13.981	<.001
TIME10	30 - 0	7.935	0.560	6.83	8.96661	148.00	14.170	<.001
TIME11	45 - 0	8.328	0.560	7.22	9.38771	148.00	14.872	<.001
TIME12	60 - 0	8.434	0.560	7.25	9.53997	148.00	15.060	<.001
TIME13	90 - 0	8.796	0.560	7.70	9.85953	148.00	15.707	<.001
TIME14	120 - 0	8.892	0.560	7.74	9.99030	148.00	15.879	<.001
TIME15	150 - 0	9.119	0.560	7.93	10.21908	148.00	16.284	<.001
TIME16	180 - 0	9.198	0.560	8.04	10.31865	148.00	16.426	<.001
TIME17	210 - 0	9.276	0.560	8.11	10.33428	148.00	16.564	<.001

Table S24. GV release - Parameter Estimates (Fixed coefficients).

Parameter Estimates (Fixed coefficients)

				95% Confidence Intervals				
Names	Effect	Estimate	SE	Lower	Upper	df	t	p
TIME18	240 - 0	9.421	0.560	8.32	10.53550	148.00	16.824	<.001
TIME19	270 - 0	9.511	0.560	8.47	10.61005	148.00	16.983	<.001
TIME20	300 - 0	9.518	0.560	8.40	10.59592	148.00	16.996	<.001
TIME21	330 - 0	9.689	0.560	8.62	10.85857	148.00	17.301	<.001
TIME22	360 - 0	9.774	0.560	8.62	10.82447	148.00	17.454	<.001
TIME23	1440 - 0	10.453	0.560	9.38	11.57213	148.00	18.666	<.001
TIME24	2880 - 0	10.713	0.560	9.53	11.79544	148.00	19.130	<.001
SAMPLE1 ✻ TIME1	(GV_GEL_1 - GV_CONTROL) ✻ (1 - 0)	-7.088	1.372	-9.73	-4.20147	148.00	-5.167	<.001
SAMPLE2 ✻ TIME1	(GV_GEL_2 - GV_CONTROL) ✻ (1 - 0)	-2.665	1.372	-5.35	0.23888	148.00	-1.943	0.054
SAMPLE1 ✻ TIME2	(GV_GEL_1 - GV_CONTROL) ✻ (2 - 0)	-7.010	1.372	-9.58	-4.39980	148.00	-5.110	<.001
SAMPLE2 ✻ TIME2	(GV_GEL_2 - GV_CONTROL) ✻ (2 - 0)	-2.060	1.372	-4.56	0.74163	148.00	-1.502	0.135
SAMPLE1 ✻ TIME3	(GV_GEL_1 - GV_CONTROL) ✻ (3 - 0)	-6.687	1.372	-9.22	-3.84376	148.00	-4.875	<.001
SAMPLE2 ✻ TIME3	(GV_GEL_2 - GV_CONTROL) ✻ (3 - 0)	-1.407	1.372	-4.04	1.24385	148.00	-1.026	0.307
SAMPLE1 ✻ TIME4	(GV_GEL_1 - GV_CONTROL) ✻ (4 - 0)	-6.315	1.372	-8.79	-3.61051	148.00	-4.603	<.001
SAMPLE2 ✻ TIME4	(GV_GEL_2 - GV_CONTROL) ✻ (4 - 0)	-1.203	1.372	-3.67	1.51634	148.00	-0.877	0.382
SAMPLE1 ✻ TIME5	(GV_GEL_1 - GV_CONTROL) ✻ (5 - 0)	-6.119	1.372	-8.58	-3.46230	148.00	-4.461	<.001
SAMPLE2 ✻ TIME5	(GV_GEL_2 - GV_CONTROL) ✻ (5 - 0)	-1.180	1.372	-3.69	1.59266	148.00	-0.860	0.391
SAMPLE1 ✻ TIME6	(GV_GEL_1 - GV_CONTROL) ✻ (10 - 0)	-5.939	1.372	-8.52	-3.20019	148.00	-4.330	<.001
SAMPLE2 ✻ TIME6	(GV_GEL_2 - GV_CONTROL) ✻ (10 - 0)	-1.141	1.372	-3.74	1.59932	148.00	-0.832	0.407
SAMPLE1 ✻ TIME7	(GV_GEL_1 - GV_CONTROL) ✻ (15 - 0)	-5.491	1.372	-7.94	-2.97870	148.00	-4.003	<.001

Table S24. GV release - Parameter Estimates (Fixed coefficients).

Parameter Estimates (Fixed coefficients)

Names	Effect	Estimate	SE	95% Confidence Intervals Lower	Upper	df	t	p
SAMPLE2 ✻ TIME7	(GV_GEL_2 - GV_CONTROL) ✻ (15 - 0)	-1.065	1.372	-3.62	1.62489	148.00	-0.777	0.439
SAMPLE1 ✻ TIME8	(GV_GEL_1 - GV_CONTROL) ✻ (20 - 0)	-5.471	1.372	-8.08	-2.77116	148.00	-3.988	<.001
SAMPLE2 ✻ TIME8	(GV_GEL_2 - GV_CONTROL) ✻ (20 - 0)	-1.009	1.372	-3.54	1.80683	148.00	-0.735	0.463
SAMPLE1 ✻ TIME9	(GV_GEL_1 - GV_CONTROL) ✻ (25 - 0)	-5.000	1.372	-7.48	-2.22665	148.00	-3.645	<.001
SAMPLE2 ✻ TIME9	(GV_GEL_2 - GV_CONTROL) ✻ (25 - 0)	-1.038	1.372	-3.39	1.77707	148.00	-0.757	0.450
SAMPLE1 ✻ TIME10	(GV_GEL_1 - GV_CONTROL) ✻ (30 - 0)	-5.060	1.372	-7.65	-2.23497	148.00	-3.689	<.001
SAMPLE2 ✻ TIME10	(GV_GEL_2 - GV_CONTROL) ✻ (30 - 0)	-0.822	1.372	-3.20	1.95829	148.00	-0.599	0.550
SAMPLE1 ✻ TIME11	(GV_GEL_1 - GV_CONTROL) ✻ (45 - 0)	-4.435	1.372	-7.11	-1.54620	148.00	-3.233	0.002
SAMPLE2 ✻ TIME11	(GV_GEL_2 - GV_CONTROL) ✻ (45 - 0)	-0.438	1.372	-2.81	2.31285	148.00	-0.319	0.750
SAMPLE1 ✻ TIME12	(GV_GEL_1 - GV_CONTROL) ✻ (60 - 0)	-4.241	1.372	-6.77	-1.61285	148.00	-3.091	0.002
SAMPLE2 ✻ TIME12	(GV_GEL_2 - GV_CONTROL) ✻ (60 - 0)	-0.525	1.372	-3.09	2.23596	148.00	-0.383	0.702
SAMPLE1 ✻ TIME13	(GV_GEL_1 - GV_CONTROL) ✻ (90 - 0)	-3.324	1.372	-5.80	-0.47714	148.00	-2.423	0.017
SAMPLE2 ✻ TIME13	(GV_GEL_2 - GV_CONTROL) ✻ (90 - 0)	-0.355	1.372	-3.00	2.41179	148.00	-0.259	0.796
SAMPLE1 ✻ TIME14	(GV_GEL_1 - GV_CONTROL) ✻ (120 - 0)	-3.171	1.372	-5.82	-0.56704	148.00	-2.312	0.022
SAMPLE2 ✻ TIME14	(GV_GEL_2 - GV_CONTROL) ✻ (120 - 0)	-0.416	1.372	-2.92	2.20938	148.00	-0.303	0.762
SAMPLE1 ✻ TIME15	(GV_GEL_1 - GV_CONTROL) ✻ (150 - 0)	-2.848	1.372	-5.39	-0.28999	148.00	-2.076	0.040
SAMPLE2 ✻ TIME15	(GV_GEL_2 - GV_CONTROL) ✻ (150 - 0)	-0.262	1.372	-2.86	2.48197	148.00	-0.191	0.849
SAMPLE1 ✻ TIME16	(GV_GEL_1 - GV_CONTROL) ✻ (180 - 0)	-2.633	1.372	-5.16	-0.00159	148.00	-1.919	0.057
SAMPLE2 ✻ TIME16	(GV_GEL_2 - GV_CONTROL) ✻ (180 - 0)	-0.449	1.372	-3.08	2.37193	148.00	-0.328	0.744
SAMPLE1 ✻ TIME17	(GV_GEL_1 - GV_CONTROL) ✻ (210 - 0)	-2.559	1.372	-5.09	0.06155	148.00	-1.866	0.064

Table S24. GV release - Parameter Estimates (Fixed coefficients).

Parameter Estimates (Fixed coefficients)

				95% Confidence Intervals				
**Names**	**Effect**	**Estimate**	**SE**	**Lower**	**Upper**	**df**	**t**	**p**
SAMPLE2 ✻ TIME17	(GV_GEL_2 - GV_CONTROL) ✻ (210 - 0)	-0.359	1.372	-2.89	2.37922	148.00	-0.261	0.794
SAMPLE1 ✻ TIME18	(GV_GEL_1 - GV_CONTROL) ✻ (240 - 0)	-2.266	1.372	-4.83	0.44179	148.00	-1.652	0.101
SAMPLE2 ✻ TIME18	(GV_GEL_2 - GV_CONTROL) ✻ (240 - 0)	-0.424	1.372	-2.96	2.32921	148.00	-0.309	0.758
SAMPLE1 ✻ TIME19	(GV_GEL_1 - GV_CONTROL) ✻ (270 - 0)	-2.171	1.372	-4.79	0.60493	148.00	-1.583	0.116
SAMPLE2 ✻ TIME19	(GV_GEL_2 - GV_CONTROL) ✻ (270 - 0)	-0.353	1.372	-3.04	2.31120	148.00	-0.258	0.797
SAMPLE1 ✻ TIME20	(GV_GEL_1 - GV_CONTROL) ✻ (300 - 0)	-2.279	1.372	-4.78	0.37608	148.00	-1.662	0.099
SAMPLE2 ✻ TIME20	(GV_GEL_2 - GV_CONTROL) ✻ (300 - 0)	-0.386	1.372	-3.01	2.55882	148.00	-0.281	0.779
SAMPLE1 ✻ TIME21	(GV_GEL_1 - GV_CONTROL) ✻ (330 - 0)	-2.000	1.372	-4.84	1.02632	148.00	-1.458	0.147
SAMPLE2 ✻ TIME21	(GV_GEL_2 - GV_CONTROL) ✻ (330 - 0)	-0.391	1.372	-3.28	2.27898	148.00	-0.285	0.776
SAMPLE1 ✻ TIME22	(GV_GEL_1 - GV_CONTROL) ✻ (360 - 0)	-2.074	1.372	-4.61	0.80907	148.00	-1.512	0.133
SAMPLE2 ✻ TIME22	(GV_GEL_2 - GV_CONTROL) ✻ (360 - 0)	-0.458	1.372	-3.05	2.18423	148.00	-0.334	0.739
SAMPLE1 ✻ TIME23	(GV_GEL_1 - GV_CONTROL) ✻ (1440 - 0)	-2.880	1.372	-5.40	-0.18745	148.00	-2.100	0.037
SAMPLE2 ✻ TIME23	(GV_GEL_2 - GV_CONTROL) ✻ (1440 - 0)	-0.610	1.372	-3.13	2.04647	148.00	-0.445	0.657
SAMPLE1 ✻ TIME24	(GV_GEL_1 - GV_CONTROL) ✻ (2880 - 0)	-3.414	1.372	-6.04	-0.62139	148.00	-2.489	0.014
SAMPLE2 ✻ TIME24	(GV_GEL_2 - GV_CONTROL) ✻ (2880 - 0)	-0.625	1.372	-3.17	2.18847	148.00	-0.455	0.649

Effect: Predictor term.
Estimate (B): Estimated effect size; expected change in the dependent variable associated with a one-unit increase in the predictor.
SE (Standard Error): Precision of the estimate; lower values indicate greater reliability.
df: Degrees of freedom associated with the t-test for the effect.
t: Test statistic indicating how far the estimate is from zero, in standard error units.
p: p-value assessing the statistical significance of the effect.

Table S25. GV release – Random components.

Groups	Name	Variance	SD	ICC
ID	(Intercept)	0.425	0.652	0.232
Residual		1.411	1.188	

Variance: Estimated variance for the random effect (e.g., subject-level variability).
SD (Standard Deviation): Square root of the variance, indicating the spread of random intercepts.
ICC (Intraclass Correlation Coefficient): Proportion of total variance explained by between-subject differences. Values closer to 1 indicate high within-subject consistency across repeated measures.

Table S26. GV release – Post hoc comparison: SAMPLE.

Comparison							
**SAMPLE**	*vs*	**SAMPLE**	**Difference**	**SE**	**t**	**df**	p_{tukey}
GV_CONTROL	-	GV_GEL_1	4.019	0.194	20.72	148	<0.001
GV_CONTROL	-	GV_GEL_2	0.786	0.194	4.05	148	<0.001
GV_GEL_1	-	GV_GEL_2	-3.233	0.194	-16.67	148	<0.001

Difference: Estimated mean difference between two levels of the factor.
SE (Standard Error): Standard error of the estimated difference.
t: Test statistic for the contrast.
df: Degrees of freedom for the comparison.
p Tukey: p-value adjusted using Tukey's method.

References

1. Baker, R.W.; Lonsdale, H.K. Controlled Release: Mechanisms and Rates. In *Controlled Release of Biologically Active Agents*; Tanquary, A.C., Lacey, R.E., Eds.; Advances in Experimental Medicine and Biology; Springer US: Boston, MA, 1974; Vol. 47, pp. 15–71 ISBN 978-1-4684-7241-7.
2. *Polymer Particles*; Okubo, M., Ed.; Advances in Polymer Science; Springer Berlin Heidelberg: Berlin, Heidelberg, 2005; ISBN 978-3-540-22923-0.
3. S K, S.; Patra, N.P.; J, S.; Rao, M.E.B. Design and Evaluation of Sustained Release Solid Dispersions of Verapamil Hydrochloride. *PCI- Approved-IJPSN* **2011**, *3*, 1252–1262, doi:10.37285/ijpsn.2010.3.4.10.
4. Rehman, F.; Khan, A.J.; Sama, Z.U.; Alobaid, H.M.; Gilani, M.A.; Safi, S.Z.; Muhammad, N.; Rahim, A.; Ali, A.; Guo, J.; et al. Surface Engineered Mesoporous Silica Carriers for the Controlled Delivery of Anticancer Drug 5-Fluorouracil: Computational Approach for the Drug-Carrier Interactions Using Density Functional Theory. *Front. Pharmacol.* **2023**, *14*, 1146562, doi:10.3389/fphar.2023.1146562.
5. Zimmer, Ł.; Belniak, P.; Szumiło, M.; Kasperek, R.; Poleszak, E. Release Kinetics of Sulfadimidine Sodium and Trimethoprim from Tablets Containing Different Excipients Prepared by Wet Granulation Method. *Curr. Issues Pharm. Med. Sci.* **2013**, *26*, 183–188, doi:10.12923/j.2084-980X/26.2/a.14.
6. Katime, I.; Mendizábal, E. Swelling Properties of New Hydrogels Based on the Dimethyl Amino Ethyl Acrylate Methyl Chloride Quaternary Salt with Acrylic Acid and 2-Methylene Butane-1,4-Dioic Acid Monomers in Aqueous Solutions. *MSA* **2010**, *01*, 162–167, doi:10.4236/msa.2010.13026.

7. Sharma, S.; Dua, A.; Malik, A. Biocompatible Stimuli Responsive Superabsorbent Polymer for Controlled Release of GHK-Cu Peptide for Wound Dressing Application. *J Polym Res* **2017**, *24*, doi:10.1007/s10965-017-1254-z.
8. Tiruneh, G.A.; Alemayehu, T.Y.; Allouche, F.K.; Reichert, J.M. Spatial Variability Modeling of Soil Fertility for Improved Nutrient Management in Northwest Ethiopia. *Arab J Geosci* **2021**, *14*, doi:10.1007/s12517-021-08814-5.
